# Exposing the Limitations of Molecular Machine Learning with Activity Cliffs

**DOI:** 10.1021/acs.jcim.2c01073

**Published:** 2022-12-01

**Authors:** Derek van Tilborg, Alisa Alenicheva, Francesca Grisoni

**Affiliations:** † Institute for Complex Molecular Systems and Dept. Biomedical Engineering, Eindhoven University of Technology, 5612AZEindhoven, The Netherlands; ‡ Centre for Living Technologies, Alliance TU/e, WUR, UU, UMC Utrecht, 3584CBUtrecht, The Netherlands; § JetBrains Research, 194100Saint Petersburg, Russia

## Abstract

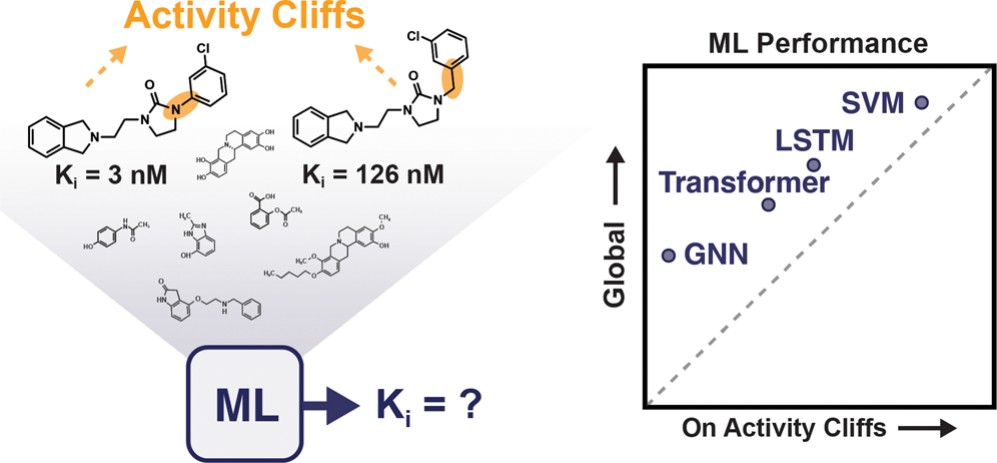

Machine learning has become a crucial tool in drug discovery and chemistry at large, *e.g.*, to predict molecular properties, such as bioactivity, with high accuracy. However, activity cliffs—pairs of molecules that are highly similar in their structure but exhibit large differences in potency—have received limited attention for their effect on model performance. Not only are these edge cases informative for molecule discovery and optimization but also models that are well equipped to accurately predict the potency of activity cliffs have increased potential for prospective applications. Our work aims to fill the current knowledge gap on best-practice machine learning methods in the presence of activity cliffs. We benchmarked a total of 24 machine and deep learning approaches on curated bioactivity data from 30 macromolecular targets for their performance on activity cliff compounds. While all methods struggled in the presence of activity cliffs, machine learning approaches based on molecular descriptors outperformed more complex deep learning methods. Our findings highlight large case-by-case differences in performance, advocating for (a) the inclusion of dedicated “activity-cliff-centered” metrics during model development and evaluation and (b) the development of novel algorithms to better predict the properties of activity cliffs. To this end, the methods, metrics, and results of this study have been encapsulated into an open-access benchmarking platform named MoleculeACE (Activity Cliff Estimation, available on GitHub at: https://github.com/molML/MoleculeACE). MoleculeACE is designed to steer the community toward addressing the pressing but overlooked limitation of molecular machine learning models posed by activity cliffs.

## Introduction

In the last decade, artificial intelligence (AI) in the form of machine learning has permeated many domains of science. The chemical sciences have particularly benefited from the AI renaissance.^1−3^ In multiple applications, machine learning has performed *on par* or even outperformed existing approaches, *e.g.*, for computer-assisted synthesis planning,^4−6^ protein structure prediction,^7,8^ and *de novo* molecular design.^9−11^ Most AI breakthroughs in chemistry have been driven by deep learning—based on neural networks with multiple processing layers.^12−14^ However, there is currently no consensus on whether deep learning models outperform simpler machine learning approaches when it comes to molecular property prediction.^15−17^ The identification of current gaps in machine and deep learning approaches would allow the development of more reliable and widely applicable models to accelerate molecule discovery.

Molecular property prediction has the principle of similarity at its heart^18^—postulating that similar compounds are likely to have similar properties. Notably, one particular exception to this principle holds great insights into the underlying structure–activity (or structure–property) relationships.^19^ Such an exception is constituted by activity cliffs^20^—pairs of structurally similar molecules that exhibit a large difference in their biological activity. Activity cliffs may cause machine learning models to remarkably mispredict the activity of certain molecules, even with an overall high model predictivity. Although generally constituting a source of “disappointment”,^20^ activity cliffs also encode valuable information for many applications^19^ (*e.g.*, hit-to-lead optimization,^21,22^ structural alert development^23^) since the large change in activity is induced by small structural changes.^24,25^ Activity cliffs are particularly relevant in the context of virtual screening, with the number of highly similar molecules in commonly used commercial libraries varying between 10,000 and 170,000 (Supporting Table S1). While numerous studies have focused on defining activity cliffs,^19,24,26,27^ their detrimental effect on machine learning models has been disproportionately underinvestigated.^25^ Arguably, models that can provide better predictions on activity cliffs are overall better, as they capture the underlying “structure–activity landscape”^20^ more accurately. Finally, although (macromolecular) structure-based approaches can aid in identifying discontinuities in the activity landscape,^28^ ligand-based methods are routinely employed “out of the box” for virtual screening without incorporating considerations on activity cliffs.

Stemming from these considerations, the presented work has a threefold goal: (1) benchmark the performance of several machine and deep learning methods on activity cliffs, (2) quantify the effect of activity cliffs on the overall performance of machine learning, and (3) identify promising approaches and future directions in the field of molecular machine learning. To this end, we compared sixteen “traditional” machine learning methods—based on human-engineered features (“molecular descriptors”^29^)—with seven deep learning approaches based on molecular strings or graphs to predict the biological activity of more than 35,000 molecules over 30 macromolecular targets. Our results highlight a generally poor performance of machine learning approaches on activity cliff compounds (particularly evident for deep learning), thereby further underscoring the relevance of assessing structure–activity “discontinuities” during model training and selection.

To further steer the community’s efforts toward the relevant topic of activity cliffs, the results of our study were encapsulated in a dedicated benchmarking platform called MoleculeACE (“Activity Cliff Estimation”). MoleculeACE complements existing benchmarks and data sets for molecular property prediction^30−33^ by providing a novel framework specifically focused on identifying activity cliffs and quantifying the corresponding model performance. MoleculeACE positions itself in a broader movement within the machine learning community^34−36^ and aims to survey the landscape of existing AI approaches systematically for molecular property prediction.^37^

## Results and Discussion

### Study Setup

#### Data Sets and Activity Cliff Definition

To ensure a comprehensive analysis of model performance, we collected and curated data on 30 macromolecular targets from ChEMBL^38^ v29 (Table 1). Acknowledging known limitations of public data, we tried to rule out the presence of significant sources of error as much as possible and curated molecules according to best practices.^39−41^ In particular, we checked for (a) the presence of duplicates, salts, and mixtures; (b) the consistency of structural annotations (*i.e.*, molecular validity and “sanity”, charge standardization, and stereochemistry definition); and (c) the reliability of the reported experimental values in terms of annotated validity, the standard deviation of multiple entries, and the presence of outliers (see Materials and Methods section). The curated collection contains a total of 48,707 molecules (of which 35,632 were unique) and mimics typical drug discovery data sets, as it (a) includes several target families relevant for drug discovery (*e.g.*, kinases, nuclear receptors, G-protein-coupled receptors, transferases, and proteases) and (b) spans different training scenarios, from small (*e.g.*, 615 molecules for Janus Kinase 1 [JAK1]) to large (*e.g*., 3657 molecules, dopamine D3 receptor [DRD3]) data sets (Table 1).

**Table 1:** Data Set Overview, with Response Type (Inhibition [Inhibitory Constant, *K*_i_] or Agonism [Half-Maximal Effective Concentration, EC_50_]), the Number of Total and Test Set Molecules (*n* and *n*_TEST_, Respectively), along with the Percentage of Total and Test Activity Cliffs (%cliff and %cliff_test_)^a^

target name	type	*n* (*n*_TEST_)	%cliff (%cliff_TEST_)
androgen receptor (AR)	*K*_i_	659 (134)	24 (23)
cannabinoid receptor 1 (CB1)	EC_50_	1031 (208)	36 (36)
coagulation factor X (FX)	*K*_i_	3097 (621)	44 (43)
delta opioid receptor (DOR)	*K*_i_	2598 (521)	37 (37)
dopamine D3 receptor (D3R)	*K*_i_	3657 (734)	39 (40)
dopamine D4 receptor (D4R)	*K*_i_	1859 (374)	38 (38)
dopamine transporter (DAT)	*K*_i_	1052 (213)	25 (25)
dual specificity protein kinase CLK4	*K*_i_	731 (149)	9 (9)
farnesoid X receptor (FXR)	EC_50_	631 (128)	39 (39)
ghrelin receptor (GHSR)	EC_50_	682 (139)	48 (49)
glucocorticoid receptor (GR)	*K*_i_	750 (152)	31 (31)
glycogen synthase kinase-3 β (GSK3)	*K*_i_	856 (173)	18 (18)
histamine H1 receptor (HRH1)	*K*_i_	973 (197)	23 (23)
histamine H3 receptor (HRH3)	*K*_i_	2862 (574)	38 (38)
janus kinase 1 (JAK1)	*K*_i_	615 (126)	7 (8)
janus kinase 2 (JAK2)	*K*_i_	976 (197)	12 (13)
kappa opioid receptor (KOR) agonism	EC_50_	955 (193)	42 (42)
kappa opioid receptor (KOR) inhibition	*K*_i_	2602 (521)	36 (36)
mu-opioid receptor (MOR)	*K*_i_	3142 (630)	35 (35)
orexin receptor 2 (OX2R)	*K*_i_	1471 (297)	52 (52)
peroxisome proliferator-activated receptor alpha (PPARα)	EC_50_	1721 (344)	41 (41)
peroxisome proliferator-activated receptor gamma (PPARγ)	EC_50_	2349 (470)	38 (38)
peroxisome proliferator-activated receptor delta (PPARδ)	EC_50_	1125 (225)	42 (42)
PI3-kinase p110-α subunit (PIK3CA)	*K*_i_	960 (193)	37 (36)
serine/threonine-protein kinase PIM1	*K*_i_	1456 (294)	33 (33)
serotonin 1a receptor (5-HT1A)	*K*_i_	3317 (666)	35 (35)
serotonin transporter (SERT)	*K*_i_	1704 (342)	35 (35)
sigma opioid receptor (SOR)	*K*_i_	1328 (267)	35 (35)
thrombin (F2)	*K*_i_	2754 (553)	36 (36)
tyrosine-protein kinase ABL1	*K*_i_	794 (161)	32 (32)

a An extensive description of the data sets can be found in Supporting Table S2.

**Figure 1:**
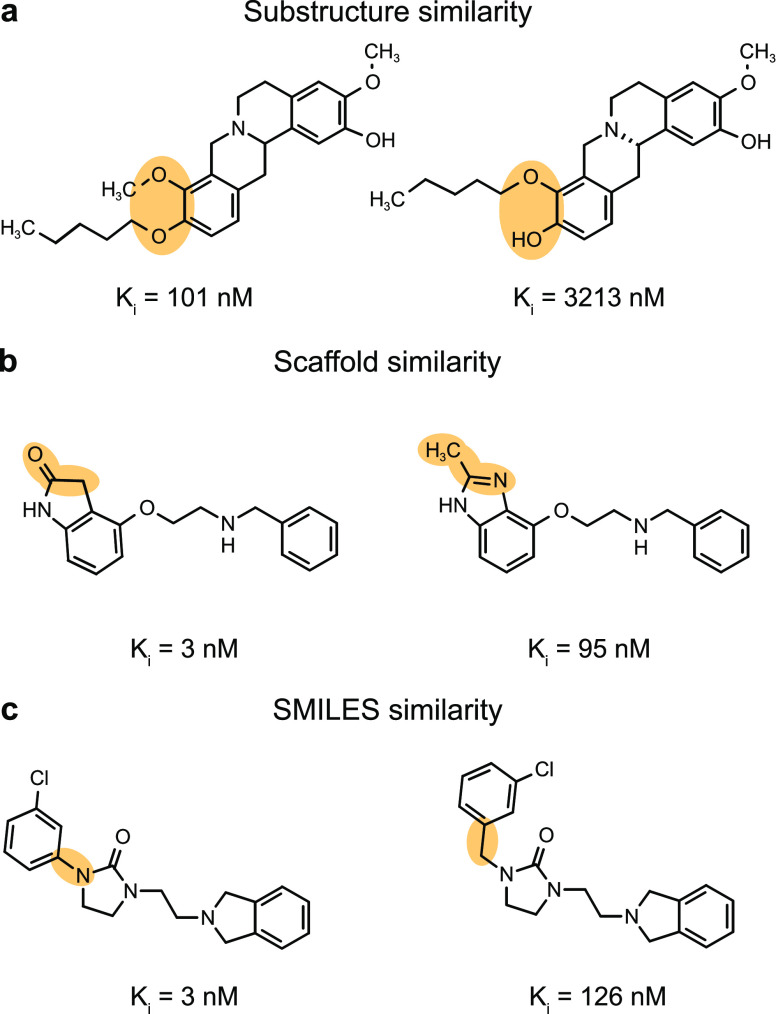
Selected examples of activity cliffs (on dopamine D3 receptor, D3R). (a) General substructure similarity (Tanimoto coefficient on ECFP). (b) Scaffold similarity that quantifies the similarity between molecular cores or scaffold decorations (Tanimoto coefficient on scaffold ECFP). (c) SMILES similarity that detects string insertions, deletions, and translocations (scaled Levenshtein distance).

For each macromolecular target, activity cliffs were identified by considering pairwise structural similarities and differences in potency. We quantified molecular similarity between any pairs of molecules belonging to the same data set with three distinct approaches:

1. *Substructure similarity*. We computed the Tanimoto coefficient^42^ on extended connectivity fingerprints^43^ (ECFPs) to capture the presence of shared radial, atom-centered substructures among pairs of molecules. This approach captures “global” differences between molecules by considering the entire set of substructures they contain (Figure 1a).
2. *Scaffold similarity*, determined by computing ECFPs on atomic scaffolds^44^ and calculating the respective Tanimoto similarity coefficient. The scaffold similarity allows identifying pairs of compounds that have minor differences in their molecular cores or differ based on their scaffold decoration (Figure 1b).
3. *Similarity of SMILES strings*, captured by the Levenshtein distance.^45^ This metric detects character insertions, deletions, and translocations (Figure 1c).

Although there is no widely accepted definition of activity cliffs^19,46,47^ and each similarity metric captures only part of the underlying “chemical reality,” these three definitions were chosen to cover different types of structural differences relevant to medicinal chemistry. Moreover, they are in line with existing literature on activity cliffs (Supporting Table S3). The so-called “chirality cliffs”^28^ were not considered in this study. Pairs of molecules that had a computed similarity larger than 90% with at least one of the three methods were considered as “highly similar” in structure. We specifically use a “soft” consensus to retain the unique properties the different similarity measures capture. Such pairs of compounds were then checked for their difference in reported potency. In agreement with previous studies,^21^ a onefold (10×) or larger difference in bioactivity (*i.e.*, on reported *K*_i_ or EC_50_ values) was used to identify activity cliff pairs. Compounds that formed at least one activity cliff pair were labeled as “activity cliff compounds”. The percentage of activity cliff compounds identified with our approach varied from 7% (JAK1) to 52% (OX2R, Table 1). Although widespread in their usage, we did not consider matched molecular pairs,^47,48^ as they almost doubled the number of cliff compounds compared to our initial approach while covering 86.6% of cliff compounds identified by our approach.

#### Data Splitting Strategy

The nature of activity cliffs complicates data splitting into training and test sets. Having high structural similarity but vastly differing bioactivities makes it infeasible to evenly distribute activity cliff molecules across sets by both their structure and activity. Besides, multiple molecules are often involved in the same activity cliff series: across all data sets, molecules have on average 2.7 ± 0.9 activity cliff “partners” identified by our approach (Supporting Table S4). In this work, we set out to ensure (a) a proportional representation of the number of activity cliff compounds in the train and test set (to avoid an over/underestimation of their effect on the performance) and (b) preserving structural similarity between training and test molecules, as previously suggested.^49^

To this end, for each data set, molecules were clustered based on substructure similarity using spectral clustering^50^ on extended connectivity fingerprints (ECFPs).^43^ For each cluster, molecules were split into a training (80%) and test set (20%) by stratified random sampling using their activity cliff label (see Materials and Methods section). This method ensured that, even in the case where all activity cliff “partners” end up in the test set (9.1 ± 5.3% of activity cliff molecules on average), highly similar molecules (in terms of substructure [0.80 ± 0.03], scaffold [0.93 ± 0.02], and SMILES [0.95 ± 0.01] similarity) are still present in the training set (Supporting Table S4).

To rule out any potential bias in favor of ECFPs, we set out to compare the similarities of different molecular descriptors in the training and test sets for each macromolecular target (see Materials and Methods section). An FDR-adjusted Mann–Whitney *U* test (α = 0.05) revealed no statistical difference between the distributions of the two sets across all descriptors and all targets. This indicates that the train–test similarity is also preserved when using different molecular descriptors.

#### Traditional Machine Learning Strategies

**Figure 2:**
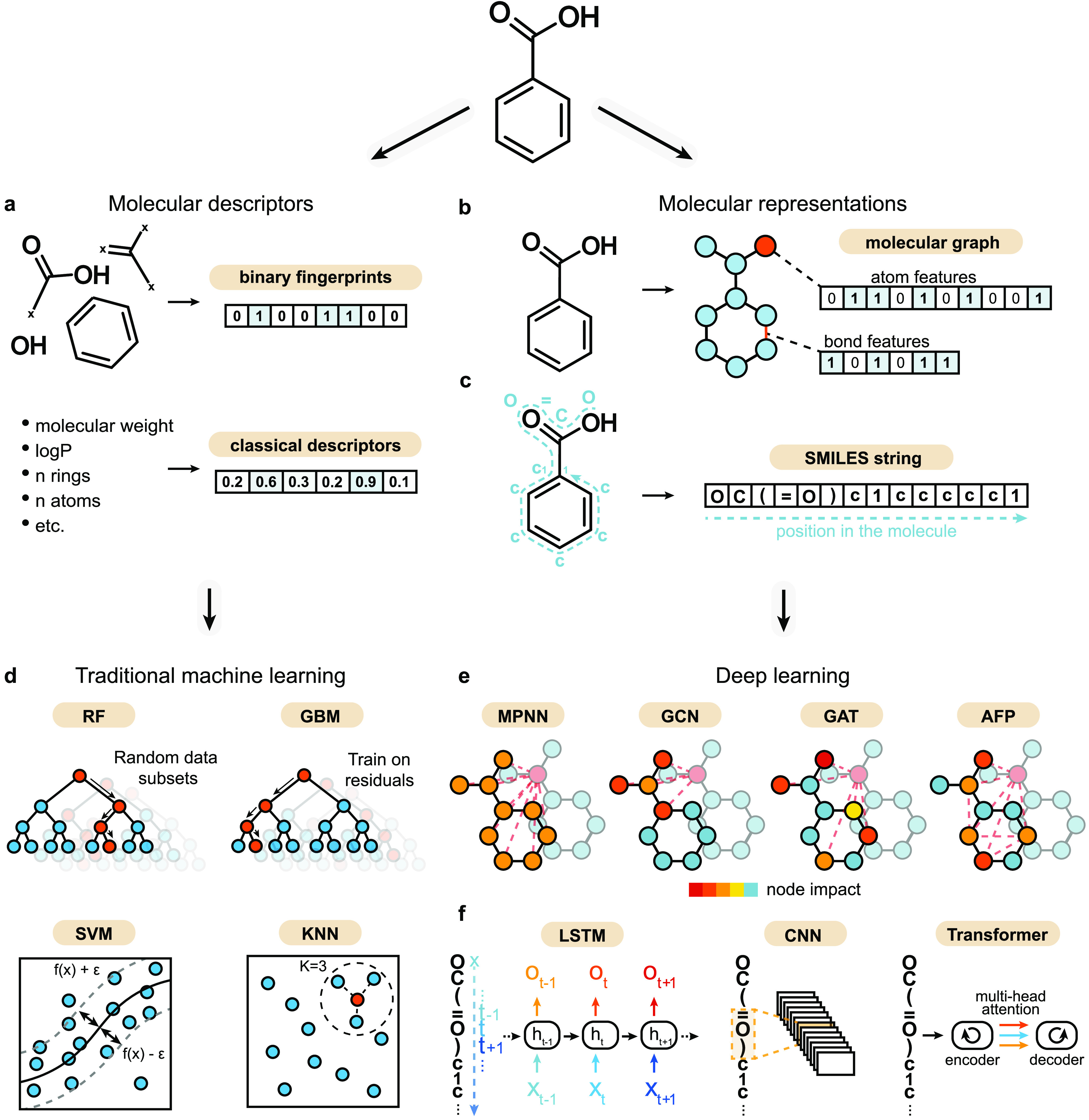
Machine learning strategies. (a) Simplified representation of molecular descriptors, which capture predefined molecular features. Both binary fingerprints and traditional molecular descriptors are used in this work. (b) Molecular graph, in which atoms are represented as nodes (with corresponding node features) and bonds are represented as edges (with corresponding edge features, if any). (c) SMILES strings, which capture two-dimensional information (atom and bond type and molecular topology) into a string. (d) Selected traditional machine learning algorithms that are trained on molecular descriptors: random forest (RF), gradient boosting (GBM), support vector regression (SVM), and *K*-nearest neighbor (KNN). (e) Deep learning methods. Four graph neural networks that can learn from molecular graphs were used: message passing neural network (MPNN), graph convolutional network (GCN), graph attention network (GAT), and attentive fingerprint (AFP). Node colors indicate the impact of other nodes during feature aggregation (indicated by dashed lines). Three SMILES-based methods that can learn from sequential data were used: long short-term memory networks (LSTM), one-dimensional (1D) convolutional neural networks (CNN), and transformers.

In this work, we considered four traditional machine learning algorithms that are commonly used for structure–activity relationship prediction (Figure 2), as follows:

1. *K-nearest neighbor* (KNN),^51^ a nonparametric approach that uses the *k* most similar training molecules to predict the response of a new molecule (as the average of the response values). Since KNN operates directly on similarity, it is expected to struggle on activity cliff molecules and was considered a baseline.
2. *Random forest* (RF),^52^ based on an ensemble of *t* distinct decision trees, each trained on various subsamples of the training set (built by bootstrapping). The molecule’s response is predicted as average over *t* predictions.
3. *Gradient boosting machine* (GBM).^53^ Like RF, this algorithm uses multiple decision trees. However, each next decision tree is optimized to minimize the residuals of the previous tree.
4. *Support vector regression* (SVM),^54^ which maps data into higher dimensions *via* a kernel function (a radial basis function in this work) to fit an optimal hyperplane to the training data.

Each algorithm was combined with four types of molecular descriptors^29^ (Figure 2), *i.e.*, human-engineered numerical features designed to capture predetermined chemical information. We explored molecular descriptors with several levels of complexity: (1) extended connectivity fingerprints^43^ (ECFPs), encoding atom-centered radial substructures^43^ in the form of a binary array; (2) Molecular ACCess System^55^ (MACCS) keys, which encode the presence of predefined substructures in a binary array; (3) weighted holistic invariant molecular (WHIM) descriptors,^56^ capturing three-dimensional molecular size, shape, symmetry, and atom distribution; and (4) 11 physicochemical properties relevant for drug-likeness^57^ (see Materials and Methods section), used as a baseline. This selection is not comprehensive (owing to the high number of existing molecular descriptors^29^), but we believe that it constitutes a good overview of different types of descriptors used in the medicinal chemistry domain.

#### Graph-Based Deep Learning

Molecular graphs are a mathematical representation of molecular topology, with nodes and edges representing atoms and chemical bonds, respectively (Figure 2b). Neural networks that can learn directly from graphs are becoming increasingly popular for molecular property prediction.^14,58−61^ In this work, we explored four neural network architectures that can directly operate on molecular graphs (Figure 2d), as follows:

1. *Message passing neural network* (MPNN).^62^ For every node in the molecular graph, information (the “message”) from neighboring nodes is aggregated by transforming it with a learnable function.
2. *Graph attention network* (GAT).^63^ Instead of a message passed across edges, this algorithm also learns attention coefficients that determine the importance of features.
3. *Graph convolutional network* (GCN),^64^ which aggregates information from neighboring nodes using a fixed convolution.
4. *Attentive fingerprint* (AFP),^59^ which uses attention mechanisms at both the atom and molecule level, allowing it to better capture subtle substructure patterns.

#### SMILES-Based Deep Learning Methods

As an additional representation, we employed the simplified molecular input line entry system (SMILES) strings,^65^ which have recently become particularly popular for *de novo* molecular design,^9−11^ and captured two-dimensional molecular information in a textual format (Figure 2c). Here, we explored three types of neural networks suitable to learn from SMILES strings:

1. *Convolutional neural network* (CNN).^66^ This neural network architecture uses a learnable convolutional filter to aggregate information from neighboring positions in a SMILES string with a sliding window approach.
2. *Long short-term memory* (LSTM)^67^ networks. LSTM—a type of recurrent neural network—can learn from string sequences by keeping track of long-range dependencies. As in a previous study,^68^ LSTM models were pretrained on SMILES obtained by merging all training sets with no repetitions (36,281 molecules) using next-character prediction before applying transfer learning for bioactivity prediction.
3. *Transformer model.* Transformers process the whole sequence at once in a graphlike manner using positional embedding to capture positional information.^69^ Transformers implement the so-called attention,^69^ which enables the model to learn which portions of the sequence are more relevant for a given task. The pretrained ChemBERTa^70^ architecture (10M compounds) was used in combination with transfer learning for bioactivity prediction.

In agreement with previous studies^66,71,72^ and thanks to the nonunivocal character of SMILES strings, we used tenfold SMILES augmentation to artificially increase the number of training samples for all approaches.

### Model Performance with Activity Cliffs

#### Traditional Machine Learning Methods

**Figure 3:**
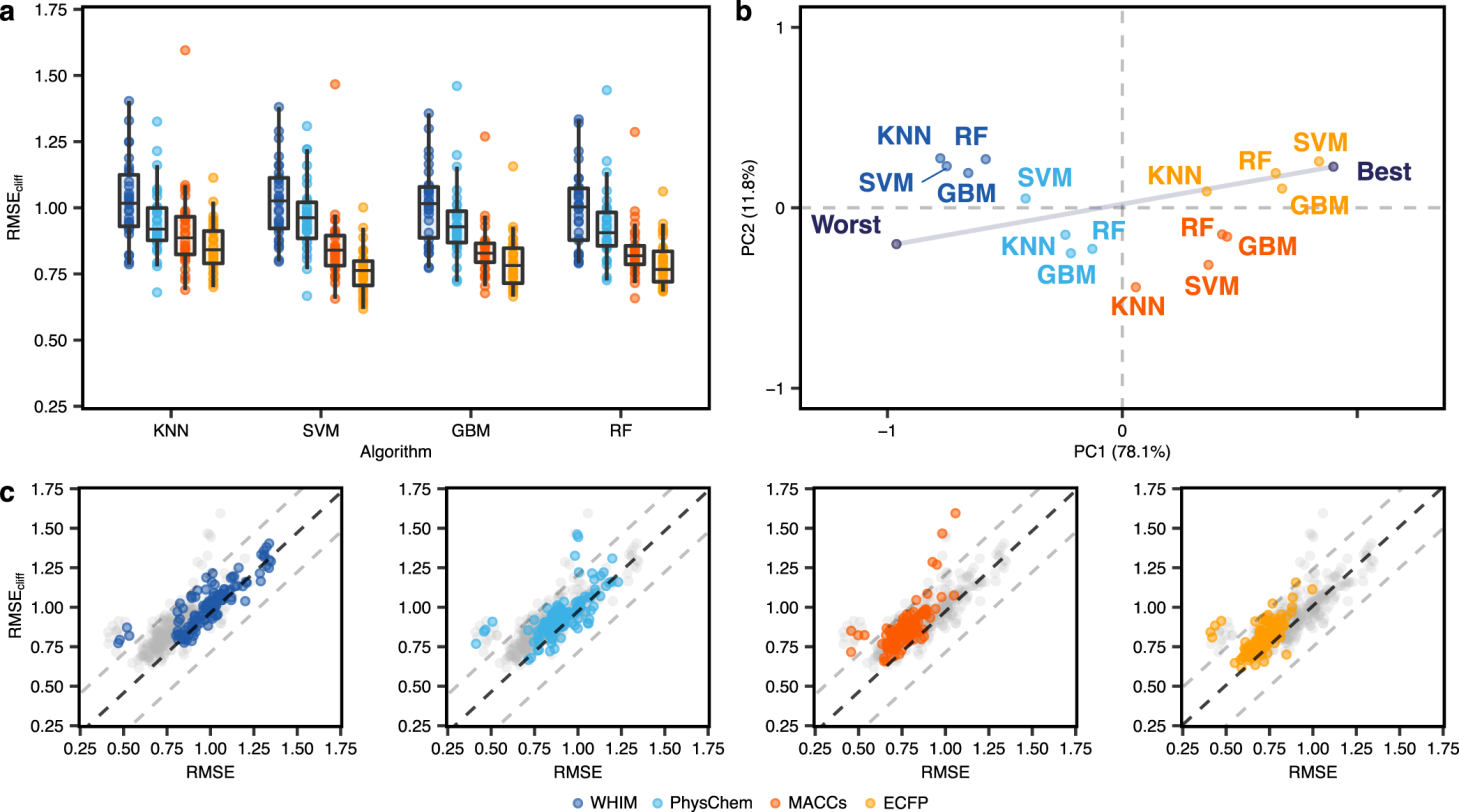
Performance of traditional machine learning methods. (a) RMSE on activity cliff compounds using different machine learning algorithms and molecular descriptors (indicated by colors). (b) Global ranking of all methods using PCA (first two principal components, PC1 and PC2), scaled between best and worst performance. Every point captures a different combination of the machine learning method and the descriptor it relied on and is obtained by considering the corresponding RMSE_cliff_ on all data sets. “Worst” and “Best” indicated the worst and best performance obtained across all data sets, respectively. Percentages represent the variance explained by each principal component. (c) Comparison between the error on activity cliff compounds (RMSE_cliff_) and the error on all compounds (RMSE) for all methods. Black dashed lines indicate RMSE = RMSE_cliff_, while gray dashed lines indicate a difference of ±0.5 log units between RMSE_cliff_ and RMSE.

First, we evaluated the ability of “traditional” machine learning approaches to predict bioactivity (expressed as pEC_50_ or p*K*_i_) in the presence of activity cliffs. The performance was quantified using the root-mean-square error on test set molecules (RMSE—the lower, the better; eq 1) and activity cliff molecules in the test set (RMSE_cliff_—the lower, the better; eq 2). Overall, large differences in predictive performance on activity cliff compounds can be observed among data sets, with RMSE_cliff_ values ranging from 0.62 to 1.60 log units (Figure 3a). This effect was also observed in the overall performance of test set molecules, with RMSE values ranging from 0.41 to 1.35 log units (Supporting Figure S1a), in line with previous works.^73−75^ Differences in performance relate mostly to the chosen molecular descriptor rather than the machine learning algorithm (*p* < 0.05, Wilcoxon rank-sum test with Benjamini–Hochberg correction, Supporting Figure S4), with ECFPs yielding the lowest average prediction error on average. Nonbinary descriptors (WHIM and physicochemical properties) performed considerably worse overall than binary fingerprints (ECFPs and MACCS), with a higher variation among data sets.

To provide a global assessment of methods across the analyzed data sets, we performed a principal component analysis (PCA) on the obtained RMSE_cliff_ values (Figure 3b and Supporting Figure S2a). PCA is a multivariate analysis technique used for data visualization and dimensionality reduction, which linearly combines the original variables into new orthogonal variables (principal components), sorted by the variance they explain. To enhance the interpretability, rows capturing the best and worst RMSE_cliff_ for each data set were added to stretch the PCA results along the direction of the best and worst results as in previous studies.^76,77^ This PCA allows considering each method on a data set-basis and to account for the presence of targets more difficult to “model”. The closer a method is to the “best” point, the better its overall performance. The higher the orthogonal deviation from the best-worst line, the higher the variability of a method’s performance based on the data set. For instance, methods based on MACCS fingerprints show a higher dependency on the chosen targets than those based on ECFPs. KNN methods show the highest dependency on the chosen target overall. Our analysis confirms the higher impact of molecular descriptors than the chosen machine learning algorithm on the model performance.^78,79^ SVM coupled with ECFPs resulted in the best method on activity cliffs on average, in agreement with a previous study.^80^ However, no statistical difference was found between SVM, GBM, or RF coupled with ECFPs (Wilcoxon rank-sum test, Supporting Figure S4). In the case of our results, however, the superior performance of ECFPs is somewhat surprising, given that they were used for the definition of activity cliffs (criteria 1 and 2, Figure 1a), which was expected to introduce an unfavorable bias.

To further investigate the relevance of considering activity cliffs for model assessment, we compared RMSE_cliff_ with the overall error on the test set molecules (Figure 3c and Supporting Figure S3). As expected, activity cliff compounds tend to yield higher prediction errors, regardless of the considered approach.^81^ Although in most of the cases RMSE and RMSE_cliff_ are highly correlated (*r* = 0.81 on average), the model performance on activity cliff compounds might be overestimated when considering RMSE alone, up to 0.54 log units. For instance, SVM coupled with ECFP descriptors—resulting in the best performance on average—ranged greatly in its ability to handle activity cliffs. While the mean difference between RMSE and RMSE_cliff_ for this method was only 0.094 log units, large differences were observed in certain data sets (*e.g*., up to 0.39 log units for the JAK1 receptor). This underscores that strategies with a low overall prediction error might not necessarily be the best ones at handling activity cliffs, thereby hampering their potential for prospective applications.

#### Deep Learning Methods

**Figure 4:**
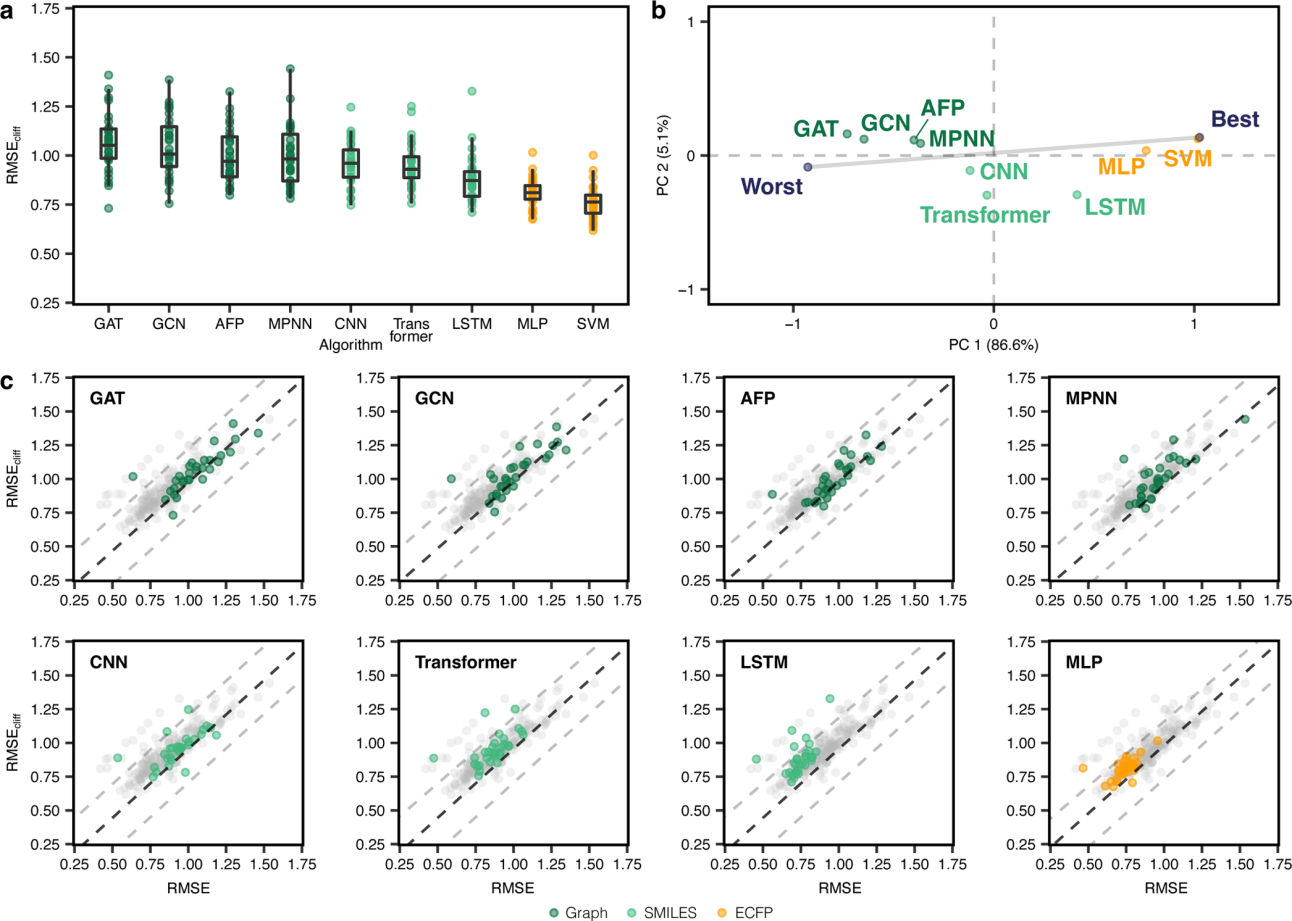
Performance of deep learning methods. (a) RMSE on activity cliff compounds on different deep learning strategies. SVM is reported as a reference. (b) Global ranking of all methods using PCA (first two principal components, PC1 and PC2), scaled between best and worst performance. Every point captures the performance of a different machine learning approach obtained by considering the corresponding RMSE_cliff_ on all data sets. “Worst” and “Best” indicated the worst and best performance obtained across all data sets, respectively. Percentages indicate the explained variance by each principal component. (c) Prediction error on activity cliff compounds (RMSE_cliff_) compared to all compounds (RMSE) for all methods.

In contrast to traditional machine learning algorithms, neural networks allow bypassing human-constructed molecular descriptors and can learn directly from “unstructured” representations of chemical structures. Deep learning approaches trained on either graphs or SMILES strings were compared with (a) a multilayer perceptron (MLP) based on ECFPs and (b) the best-performing traditional machine learning method (SVM with ECFP fingerprints), both serving as a reference point (Figure 4a).

Transfer learning^82^—applying a models’ previously learned knowledge to a new, related problem by further training—was applied to the LSTM and transformer models in agreement with previous studies.^68,70,83,84^ In a preliminary analysis, we explored transfer learning approaches for graph neural networks using self-supervision (context prediction,^85^ infomax,^86^ edge prediction,^87^ and masking^85^). Since, in line with a recent study,^88^ no approach yielded a notable increase in predictive performance, we did not consider transfer learning further for graph neural networks. When comparing the performance of all tested deep learning methods, we found large differences in predictive performance across data sets—like with traditional machine learning approaches—with RMSE_cliff_ values ranging from 0.68 to 1.44 log units (Figure 4a). Among the graph-based neural networks, MPNN models resulted in the lowest error on activity cliff compounds on average, although no differences were statistically significant (Wilcoxon rank-sum test with Benjamini–Hochberg correction, Supporting Figure S4). SMILES-based methods outperformed graph-based methods on average, with LSTM models outperforming all other deep learning methods, including the SMILES-based CNN and transformer models. For CNNs, we did not implement any transfer learning strategy, which could explain their poor(er) performance compared to the other SMILES-based methods. Notably, despite transformers being pretrained on a larger corpus of SMILES strings (10M compounds^70^), they did not perform better than LSTMs, which were pretrained on 36,281 molecules only.

When inspecting the PCA performed on the obtained RMSE_cliff_ values for each target (Figure 4b), the multilayer perceptron coupled with ECFPs outperformed all other neural networks based on SMILES or graphs. This is surprising to a certain extent, considering that ECFPs and SMILES are constructed from a molecular graph. This aspect further underscores a current gap in learning efficient features from “raw” molecular representations in the small-data regimes typical of drug discovery. Compared to most traditional machine learning approaches, deep neural networks seem to fall short at picking up subtle structural differences (and the corresponding property change) that give rise to activity cliffs. Similar results were obtained when comparing graph networks for (a) feature attribution with activity cliffs,^89^ and (b) bioactivity prediction.^30^ A recent analysis on physicochemical-property cliffs highlights an opposite trend, with deep learning methods performing better than simpler machine learning approaches^90^—potentially due to the higher number of training samples (approx. 20,000 molecules).

Interestingly, no deep learning method was stable across data sets, as shown by the large deviation from the worst-best line (Figure 4b and Supporting Figure S2b). This highlights the need to evaluate the usage of such methods on a case-by-case basis.

#### Failure Modes of Machine Learning on Activity Cliffs

The systematic training and assessment of 720 machine learning models allowed us to investigate the potential “failure modes” of machine learning approaches on activity cliffs. All methods tend to struggle in the presence of activity cliffs (Figures 3 and 4). Our first analysis addressed the variation of RMSE_cliff_ across methods and data sets in search of causes of poor performance. Although small-data regimes are known to affect the performance of machine and deep learning methods, no correlation was found between the number of molecules in the training set and the prediction error on activity cliffs (Supporting Figure S5). Furthermore, no relationship between the percentage of activity cliff compounds in the data and model performance was found, except for differences between RMSE and RMSE_cliff_. This relates to the fact that the higher the percentage of activity cliffs, the more the RMSE_cliff_ values (computed on a subset of molecules, eq 2) will approach RMSE values (Supporting Figure S6). At the same time, the drug target family did not seem to affect RMSE_cliff_ either (Supporting Figure S7), further highlighting the difficulties in forecasting the performance of machine learning on activity cliffs.

**Figure 5:**
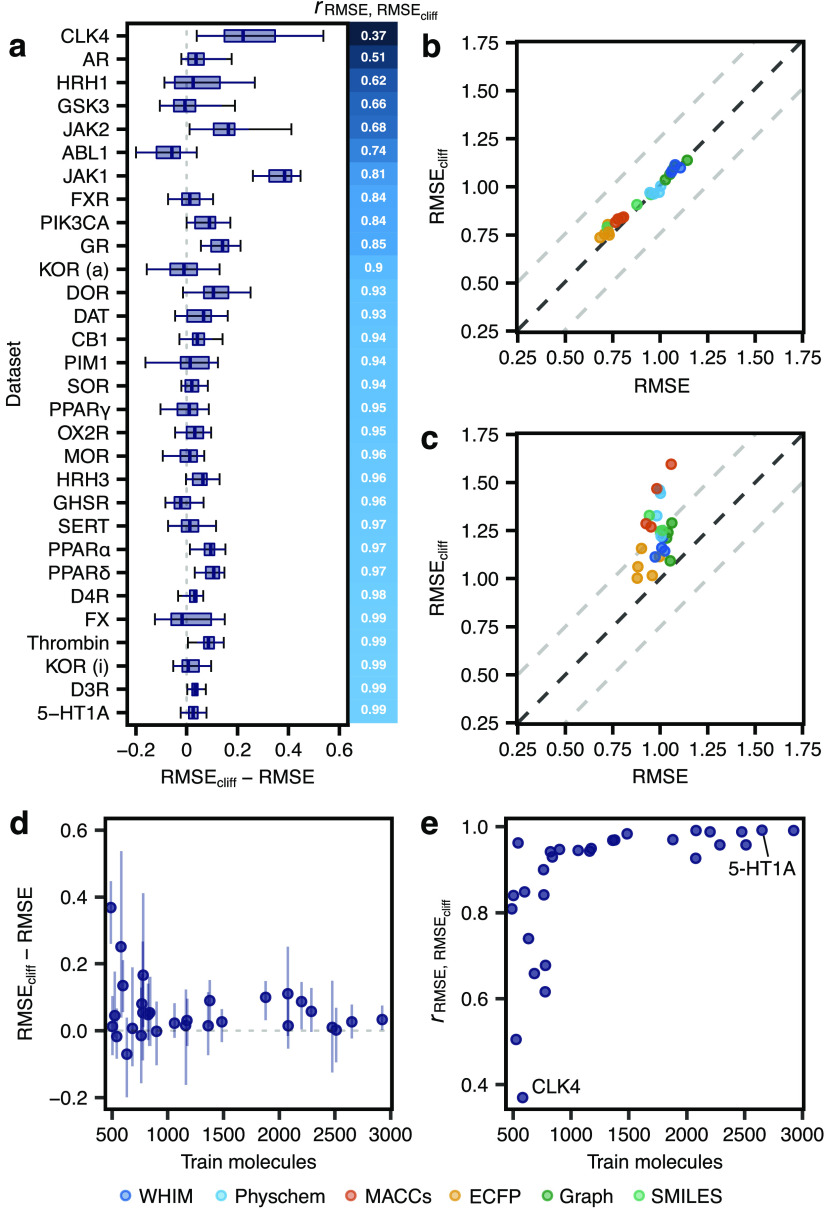
Comparing overall model performance and performance on activity cliff compounds. (a) Method-wide differences between overall RMSE and RMSE_cliff_ for all targets ordered by Pearson correlation (*r*) between RMSE and RMSE_cliff_. Error bars indicate the lowest and highest RMSE_cliff_. (b) Comparison between RMSE and RMSE_cliff_ of all methods on 5-HT1A. (c) Comparison between RMSE and RMSE_cliff_ of all methods on CLK4. (d) Effect of the number of training molecules on the difference between RMSE and RMSE_cliff_. Error bars indicate the lowest and highest RMSE_cliff_. (e) Relationship between the number of training molecules and the Pearson correlation (*r*) of RMSE and RMSE_cliff_.

We then compared the overall prediction error (RMSE on test set molecules) with the performance on activity cliffs (RMSE_cliff_ on test set molecules). While RMSE and RMSE_cliff_ tend to correlate to a high degree (*r* > 0.70 for 25 data sets out of 30, Figure 5a), we observed large case-by-case variations. In most cases, the difference between RMSE_cliff_ and RMSE is similar among methods (Figure 5a,b). This implies that, when choosing a method for its overall error on test set molecules, the performance on activity cliff compounds will be implicitly accounted for. However, for some targets (*e.g.*, CLK4), methods with comparable RMSE scores can exhibit large differences in RMSE_cliff_ scores (Figure 5c). This indicates that, in these specific cases, choosing a model based on only RMSE might lead to poor prospective performance, *e.g.*, for hit-to-lead optimization or virtual screening in the presence of congeneric compounds (Supporting Table S1). These “islands” of poor performance on activity cliffs were observed across the whole spectrum of machine learning strategies, independently of the reported average performance.

To better elucidate the “drivers of failure” on activity cliffs, we investigated the effect of the training set size on (a) the difference between predictivity on the entire test set and on activity cliffs only (RMSE_cliff_ – RMSE) and (b) the correlation between the overall performance (RMSE) and the performance on activity cliffs (RMSE_cliff_). The absolute difference between RMSE and RMSE_cliff_ does not correlate with the number of training molecules (*r* = −0.15, Figure 5d). However, the number of training molecules is an important factor in determining the correlations between RMSE and RMSE_cliff_ (Figure 5e). Data sets containing a sufficient number of training molecules (*e.g.*, larger than 1000) showed a high correlation between RMSE and RMSE_cliff_ (*r* > 0.80). In other words, if the number of training molecules increases, the “relative difficulty” of predicting bioactivity on activity cliff molecules decreases. This implies that, with a sufficient number of training molecules, optimizing RMSE alone will improve RMSE_cliff_, too. However, the problem of determining the targets on which RMSE_cliff_ will be suboptimal remains, especially in small-data regimes, further underscoring the relevance of implementing activity-cliff-related evaluation approaches. Moreover, these results corroborate the need to develop more efficient machine and deep learning models for low-data regimes.

#### Bringing It All Together: The MoleculeACE Benchmark and Future Applications

Our results and systematic analyses expose current limitations of molecular machine learning and motivate the use of dedicated metrics and tools for assessing the model performance on activity cliffs, especially in low-data regimes. Hence, we collected the modeling and assessment strategies of this study into a dedicated, “activity-cliff-centered” benchmark tool, called MoleculeACE (available at: https://github.com/molML/MoleculeACE). All data sets and scripts to replicate this study can be found here as well. MoleculeACE integrates standardized data processing for molecular bioactivity data, a comprehensive approach to quantifying activity cliffs, and the tailored performance evaluation strategies presented in this work. Thanks to its modular character, MoleculeACE will allow researchers to

1. *systematically benchmark a model’s performance* on activity cliffs compounds (*e.g.*, using different machine learning approaches or including additional molecular descriptors), in comparison with well-established machine and deep learning methods;
2. *evaluate the deck of chosen models on a new data set* not included in our benchmark, thanks to the data collection and curation pipeline; and
3. *further expand the definition of activity cliffs*(91−93) based on specific use cases.^19^ It is possible to use custom thresholds for potency differences and structural similarity (*e.g.*, matched molecular pairs, which are already supported) in determining cliff compounds. As this work relies on public bioactivity data, which might be affected by undetectable experimental noise^81,94^ (despite the best data curation efforts), we hope in the future to also see applications of MoleculeACE on more homogeneous data, *e.g.*, in terms of use *in vitro* assays and assay conditions.

We envision that MoleculeACE, along with the results of this benchmark study, will incentivize machine learning researchers to consider the crucial topic of activity cliffs in model evaluation and development pipelines. We envision that MoleculeACE will serve as a platform for the wider community to develop models that can more accurately capture complex structure–activity landscapes and ultimately boost the capabilities of machine learning for molecule discovery.

## Conclusions and Outlook

While machine learning is increasingly often employed for early drug discovery, the topic of activity cliffs has received only limited attention from the scientific community. As shown by our results, not only do machine learning strategies struggle with activity cliffs compared to their overall performance but also deep learning methods are particularly challenged by the presence of such compounds. Approaches based on human-engineered molecular descriptors resulted in outperforming deep learning based on graphs or SMILES, with no machine learning strategy being consistently better at handling activity cliffs compared to their absolute performance. Our results corroborate previous evidence showing that deep learning methods do not necessarily hold up against simpler machine learning methods (yet) for drug discovery purposes.^15−17^ Although our analysis does not allow us to identify mechanistic causes of the performance gap with activity cliffs, we speculate that current molecular representations and corresponding representation learning algorithms might not capture complex structure–activity information well enough.^95,96^ We envision the development of deep learning strategies that are (a) more efficient in low-data scenarios (*e.g.*, self-supervised learning^97^) and (b) better-suited to capture structure–activity “discontinuities” to be key for future prospective applications. Structure-based deep learning approaches^28,98,99^ (considering the structure of the macromolecular target in addition to ligand information) might be key to filling current performance gaps due to activity cliffs. However, to date, there is no consensus on the benefit of including structural information in machine learning for bioactivity prediction,^100^ potentially due to undesirable bias in existing databases.^100−102^

In the framework of our study design, the model’s performance on activity cliff compounds resulted in being highly data set-dependent, especially for deep learning methods in low-data scenarios. Although the overall prediction error often approximates the performance on activity cliffs, “islands” of poor performance on activity cliffs exist when different strategies are compared on the same data set. These results highlight the importance of evaluating machine learning models for their performance on activity cliffs, especially when prospective applications are envisioned (*e.g.*, virtual screening).^103^

To facilitate such an “activity cliff-centered” model evaluation and development, we developed MoleculeACE. By estimating a model’s performance in the presence of activity cliffs alongside regular performance, MoleculeACE has the goal of incentivizing researchers in molecular machine learning to consider the long-standing issue of activity cliffs fully. Models that can accurately predict the effects of subtle structural changes on molecular properties will ultimately give rise to more effective hit-to-lead optimization and the identification of activity cliffs during lead optimization. We envision these improvements as key to propelling the potential of deep learning in drug discovery and beyond.

## Materials and Methods

### Data Curation

#### Data Collection and Preparation

For each macromolecular target, compound bioactivity values were collected from ChEMBL^101^ v29 *via* the “ChEMBL webresource” client (*Homo sapiens*). Molecules in the form of canonical SMILES strings were sanitized using RDKit^104^ v. 2020.09.5^104^ with default settings and neutralized if charged. Compounds with failed sanitization, annotated in the form of salts, and/or with doubtful data validity (as in the “data_validity_comment” entry of ChEMBL) were removed (4.74% on average). For each unique SMILES string, experimental bioactivity data (*i.e.*, *K*_i_ or EC_50_ values [nM]) were collected. Dixon’s Q test^105^ was used to detect the presence of outliers among multiple annotations of a given molecule (α = 0.05, 0.78% of molecules on average). The mean *K*_i_ or EC_50_ value for each molecule was computed and subsequently converted into pEC_50_/p*K*_i_ values (as the negative logarithm of molar concentrations). If the standard deviation of the multiple annotations used to compute the average was above 1 log unit, the corresponding molecule was removed (4.33% on average). To rule out errors due to inconsistent annotation of stereochemistry, pairs of compounds having different canonical SMILES but identical ECFPs were removed (9.74% on average).

#### Molecular Descriptors’ Calculation

Molecular descriptors were computed from canonicalized SMILES strings using RDkit v. 2020.09.5.^104^ (a) Extended connectivity fingerprints (ECFPs)^43^ were computed with a length of 1024 bits and a radius of 2 bonds. (b) MACCS keys,^55^ with a length of 166, were computed with default settings. (c) Weighted holistic invariant molecular (WHIM) descriptors^103^ (114 descriptors) were computed on the minimum energy conformers generated with experimental-torsion knowledge distance geometry^106^ and MMFF94^107^ force field optimization. (d) “Physicochemical descriptors” included 11 properties of drug-likeness, *i.e.*, molecular weight; predicted octanol–water partitioning coefficient;^108^ molar refractivity; topological polar surface area; formal charge; and the number of hydrogen bond donors, hydrogen bond acceptors, rotatable bonds, atoms, rings, and heavy atoms. Real-valued descriptors were standardized by Gaussian normalization using the training data mean and standard deviation values.

#### Detection of Activity Cliffs

Pairs of structurally similar molecules were detected with three approaches: (a) *substructure similarity*, computed via the Tanimoto coefficient on ECFP; (b) *scaffold similarity*, calculated on the ECFP of molecular graph frameworks^44^ (Tanimoto coefficient); and (c) (canonical) *SMILES similarity*, computed using the Levenshtein distance^106,109^ (scaled and subsequently converted into “1-distance”). Pairs of compounds having a computed similarity equal to or larger than 0.9 according to at least one of these metrics were checked for the fold difference in their respective bioactivity (in nM units). Pairs of highly similar compounds showing more than tenfold difference in their respective bioactivity were considered activity cliffs.

#### Train/Test Splitting

For each target, molecules were clustered by their molecular structure (described as ECFP) into five clusters using spectral clustering^107^ implemented with sklearn v. 1.0.2^110^ (using a Gaussian kernel and a precomputed affinity matrix of Tanimoto distances). For each cluster, 80% of molecules were assigned to the training data and 20% were assigned to the test data by stratified splitting (using their belonging to at least one activity cliff pair [“yes”/“no”] as a label).

#### Descriptor Similarity between Training and Test Sets

Similarity among molecular descriptors in the training set was calculated as the mean distance of each molecule in the training set to its five nearest neighbors in the training set. The similarity between molecular descriptors of each molecule in the test set was calculated for the five nearest neighbors in the train set. Graph representations were not considered, as computing graph distances is nontrivial and ECFPs are directly related to molecular graphs. A Mann–Whitney *U* test, corrected for a false discovery rate of 0.05, was performed using SciPy v. 1.8.1.^111^

#### Molecular Graph Featurization

For all methods, atom features were encoded as follows. (a) One-hot-encoded properties included atom type, orbital hybridization, atomic vertex degree, aromaticity, and ring membership. (b) Numerically encoded properties included atomic weight, partial charge (Gasteiger–Marsili^112^), number of valence electrons, and number of bound hydrogens. The atomic weight and partial charge were scale-transformed *via* a sigmoidal function. For MPNN and AFP architectures, bond features were included, *i.e.*, with bond type and conjugation (one-hot-encoded).

### Model Implementation

#### Hyperparameter Optimization

Hyperparameter optimization was performed with Bayesian optimization using a Gaussian process (method-based specifics are mentioned below). For all models, a maximum of 50 hyperparameter combinations were evaluated using fivefold cross validation.

#### Traditional Machine Learning Algorithms

KNN, SVM, GBM, and RF regression models were implemented using sklearn v. 1.0.2.^110^ For each approach, the model hyperparameters were optimized as follows: (a) KNN, optimization of the number of neighbors (*k*), *k* = [3, 5, 11, 21]; (b) SVM, optimization of the kernel coefficient (γ) and regularization parameter (*C*), γ = [1 × 10^–6^, 1 × 10^–5^, 1 × 10^–4^, 1 × 10^–3^, 1 × 10^–2^, or 1 × 10^–1^] and *C* = [1, 10, 100, 1000, 10,000]; (c) GBM, optimization of the number of boosting stages (*n*_b_) and maximal model depth (*m*_d_), *n*_b_ = [100, 200, 400] and *m*_d_ = [5, 6, 7]; and (d) RF, number of decision trees (*t*), *t* = [100, 250, 500, 1000].

#### Graph Neural Networks

All regression models were implemented using the PyTorch Geometric package v. 2.0.4.^113^ In MPNN, GCN, and GAT models, global pooling was implemented with a graph multiset transformer^114^ using eight attention heads, followed by a fully connected prediction head. For all models, we optimized the learning rate (lr), lr = [5 × 10^–4^, 5 × 10^–5^, or 5 × 10^–6^]. The following hyperparameters were optimized:

(a) GCN, hidden atom features (*h*_a_), number of convolutional layers (*n*_c_), hidden multiset transformer nodes (*h*_t_), hidden predictor features (*h*_p_), *h*_a_ = [32, 64, 128, 256, 512], *n*_c_ = [1, 2, 3, 4, 5], *h*_t_ = [64, 128, 256, 512], *h*_p_ = [128, 256, 512];
(b) GAT, the hyperparameter search space used for GCN models and the use of GATv1^112^ or GATv2^115^ convolutions;
(c) MPNN, hidden atom features (*h*_a_), hidden edge features (*h*_e_), number of message passing steps (*s*_m_), hidden multiset transformer nodes (*h*_t_), hidden predictor features (*h*_p_), *h*_a_ = [32, 64, 128, 256], *h*_e_ = [32, 64, 128, 256], *s*_m_ = [1, 2, 3, 4, 5], *h* = [64, 128, 256, 512], *h*_p_ = [128, 256, 512]; and
(d) AFP, number of attentive layers (*n*_a_), timesteps (*n*_t_), number of hidden predictor features (*h*_p_), *n*_a_ = [1, 2, 3, 4, 5], *n*_t_ = [1, 2, 3, 4, 5], *h*_p_ = [32, 64, 128, 256].

All models were trained for 300 epochs using early stopping with a patience of ten epochs.

#### Feed-Forward Neural Network

A multilayer perceptron was implemented using Pytorch v. 1.11.0.^116^ It was optimized for (a) the learning rate (lr = [5 × 10^–4^, 5 × 10^–5^ 64, 5 × 10^–6^]), (b) the number of hidden features (*n*_*h*_ = [256, 512, 1024]), and (c) the number of layers (*n*_*l*_) = [1, 2, 3, 4, 5]. Models were trained for 500 epochs using early stopping with a patience of 10 epochs.

#### SMILES-Based Models

SMILES strings were encoded as one-hot vectors. SMILES strings longer than 200 characters were truncated (0.71% on average). Tenfold data augmentation was applied to all SMILES-based methods using a maximum of nine extra noncanonical SMILES strings for every SMILES string in the data set. Noncanonical SMILES strings were generated using RDKit.^104^

(a) LSTM models were pretrained on SMILES obtained by merging all training sets with no repetitions (36,281 molecules) using next-character prediction as in a recent study.^68^ The network was composed of four layers comprising 5,820,515 parameters (layer 1, batch normalization; layer 2, LSTM with 1024 units; layer 3, LSTM with 256 units; layer 4, batch normalization). We used the Adam optimizer with a learning rate of 10^–4^ for 100 epochs. Regression models were then obtained by transfer learning (with weight freezing for layer no. 2) for 100 epochs with a regression head.
(b) 1D CNNs were adapted from a recent study.^66^ We used a single 1D convolutional layer with a step size equal to 1, followed by a fully connected layer, with training for 500 epochs. It was optimized for the learning rate (lr), the number of hidden features in the fully connected layer (*n*_h_), and convolution kernel size (*n*_k_), lr = [5 × 10^–4^, 5 × 10^–5^,^64^ 5 × 10^–6^], *n*_h_ = [128, 256, 512, 1024], *n*_k_ = [4, 8, 10].
(c) Transformer models and the corresponding SMILES tokenization were based on the ChemBERTa^114^ architecture. We used the pretrained ChemBERTa model weights based on 10M compounds from PubChem.^117^ We fine-tuned the model by freezing its weights and replacing the final pooling layer with a regression head with one fully connected layer and trained for 100 epochs. We used the Adam optimizer with a learning rate of 5 × 10^–4^. For all methods, we used early stopping with a patience of ten epochs.

#### Performance Evaluation

The overall model performance was quantified via the root-mean-square error (RMSE) computed on the bioactivity values (*i.e.*, p*K*_i_ or pEC_50_), as follows (eq 1)
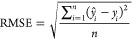
1where *ŷ*_*i*_ is the predicted bioactivity of the *i*th compound, *y_i_* is the corresponding experimental value, and *n* represents the number of considered molecules.

The performance on activity cliffs compounds was quantified by computing the root-mean-square error (RMSE_cliff_) only on compounds that belonged to at least one activity cliff pair, as follows (eq 2)
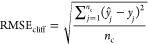
2where *ŷ*_*j*_ is the predicted bioactivity of the *j*th activity cliff compound, *y*_*j*_ is the corresponding experimental value, and *n*_c_ represents the total number of activity cliff compounds considered. *R*^2^ and *Q*^2^ metrics, or normalized RMSE values, were not considered to avoid the introduction of undesired biases related to the different range of the training/test set responses across data sets.^118,119^

## Notes

The MoleculeACE benchmark tool, together with the Python code to replicate and extend our study, is freely available on GitHub at the following URL: https://github.com/molML/MoleculeACE.The curated data sets are available at the following URL: https://github.com/molML/MoleculeACE/tree/main/MoleculeACE/Data/benchmark_data.

## Supporting Information Available

Presence of highly similar compounds in commercially available libraries (Table S1); data set overview (Table S2); activity cliff definitions across different published studies (Table S3); training/test set analysis (Table S4); overall performance of machine learning methods on all targets (Figure S1); PCA loadings of all methods (Figure S2); relative prediction error of activity cliff compounds (Figure S3); statistical differences between the RMSE_cliff_ values obtained by different machine learning strategies (Figure S4); relationship between the number of training molecules on RMSE_cliff_ (Figure S5); relationship between the fraction of activity cliff compounds and model performance (Figure S6); relationship between drug target classes and RMSE_cliff_ (Figure S7) (PDF)

## Abbreviations

AFP: attentive fingerprint
CNN: convolutional neural network
ECFP: extended connectivity fingerprints
GAT: graph attention network
GBM: gradient boosting machine
GCN: graph convolutional network
KNN: *K*-nearest neighbor
LSTM: long short-term memory network
MACCS: Molecular ACCess System
MLP: multilayer perceptron
MPNN: message passing neural network
RF: random forest
RMSE: root-mean-square error
SMILES: simplified molecular input line entry system
SVM: support vector machine
WHIM: weighted holistic invariant molecular
