# Correction to “Exposing the Limitations of Molecular Machine Learning with Activity Cliffs”

**DOI:** 10.1021/acs.jcim.3c00423

**Published:** 2023-03-30

**Authors:** Derek van Tilborg, Alisa Alenicheva, Francesca Grisoni

The sentences:

- early stopping with a patience of ten epochs. (pages 5947, 5948)
- early stopping with a patience of 10 epochs. (page 5947)

should be:

- early stopping if no improvement was obtained after ten non-consecutive epochs.

Using a patience of 10 epochs (as erroneously stated previously) does not affect the conclusions of the paper. The corresponding results can be found in the dedicated GitHub repository at the following URL: https://github.com/molML/MoleculeACE.

