# Correction to “Exposing the Limitations of Molecular Machine Learning with Activity Cliffs”

**DOI:** 10.1021/acs.jcim.3c01576

**Published:** 2023-10-18

**Authors:** Derek van Tilborg, Alisa Alenicheva, Francesca Grisoni

After publication, it came to our attention that a software bug affected the splitting between training and test sets, due to incorrect labeling of certain activity cliffs pairs. This document reports the updated Table 1 and Figures 3–5 after model retraining on the corrected data. The same findings and conclusions as the original paper were observed. The updated code (with a detailed record of the corrections), data and Supporting Information can be found at: https://github.com/molML/MoleculeACE.

For additional clarity, in Table A herein, we report the correction to all in-text references to the revised table and figures whenever values differ.

**Table 1:** (Corrected) Data Set Overview, with Response Type (Inhibition [Inhibitory Constant, *K*_i_] or Agonism [Half-Maximal Effective Concentration, EC_50_]), the Number of Total and Test Set Molecules (*n* and *n*_TEST_, Respectively), along with the Percentage of Total and Test Activity Cliff (%cliff and %cliff_TEST_) [Corrections Highlighted in Bold]

target name	type	*n*(*n*_TEST_)	%cliff (%cliff_TEST_)
androgen receptor (AR)	*K*_i_	659 (134)	2**5** (2**4**)
cannabinoid receptor 1 (CB1)	EC_50_	1031 (208)	36 (3**7**)
coagulation factor X (FX)	*K*_i_	3097 (621)	4**8** (4**8**)
delta opioid receptor (DOR)	*K*_i_	2598 (521)	3**9** (3**9**)
dopamine D3 receptor (D3R)	*K*_i_	3657 (73**3**)	**44** (4**4**)
dopamine D4 receptor (D4R)	*K*_i_	18**65** (374)	**40** (**40**)
dopamine transporter (DAT)	*K*_i_	1052 (213)	25 (2**6**)
dual specificity protein kinase CLK4	*K*_i_	731 (149)	9 (9)
farnesoid X receptor (FXR)	EC_50_	631 (128)	39 (39)
ghrelin receptor (GHSR)	EC_50_	682 (139)	**52** (**53**)
glucocorticoid receptor (GR)	*K*_i_	750 (152)	3**3** (3**4**)
glycogen synthase kinase-3 β (GSK3)	*K*_i_	856 (173)	1**9** (18)
histamine H1 receptor (HRH1)	*K*_i_	973 (197)	2**4** (2**4**)
histamine H3 receptor (HRH3)	*K*_i_	2862 (574)	**42** (**42**)
janus kinase 1 (JAK1)	*K*_i_	615 (126)	**10** (**10**)
janus kinase 2 (JAK2)	*K*_i_	976 (197)	1**7** (1**7**)
kappa opioid receptor (KOR) agonism	EC_50_	955 (193)	4**8** (4**7**)
kappa opioid receptor (KOR) inhibition	*K*_i_	260**3** (52**2**)	4**3** (4**3**)
mu-opioid receptor (MOR)	*K*_i_	3142 (630)	**41** (**41**)
orexin receptor 2 (OX2R)	*K*_i_	1471 (297)	5**4** (5**4**)
peroxisome proliferator-activated receptor alpha (PPARα)	EC_50_	1721 (34**7**)	41 (41)
peroxisome proliferator-activated receptor gamma (PPARγ)	EC_50_	2349 (47**2**)	38 (38)
peroxisome proliferator-activated receptor delta (PPARδ)	EC_50_	1125 (22**6**)	42 (42)
PI3-kinase p110-α subunit (PIK3CA)	*K*_i_	960 (193)	**42** (**42**)
serine/threonine-protein kinase PIM1	*K*_i_	1456 (294)	3**9** (3**9**)
serotonin 1a receptor (5-HT1A)	*K*_i_	3317 (666)	3**7** (3**7**)
serotonin transporter (SERT)	*K*_i_	1704 (342)	3**7** (3**7**)
sigma opioid receptor (SOR)	*K*_i_	1328 (267)	3**8** (3**9**)
thrombin (F2)	*K*_i_	2754 (553)	**40** (**40**)
tyrosine-protein kinase ABL1	*K*_i_	794 (161)	**42** (**42**)

**Figure 3:**
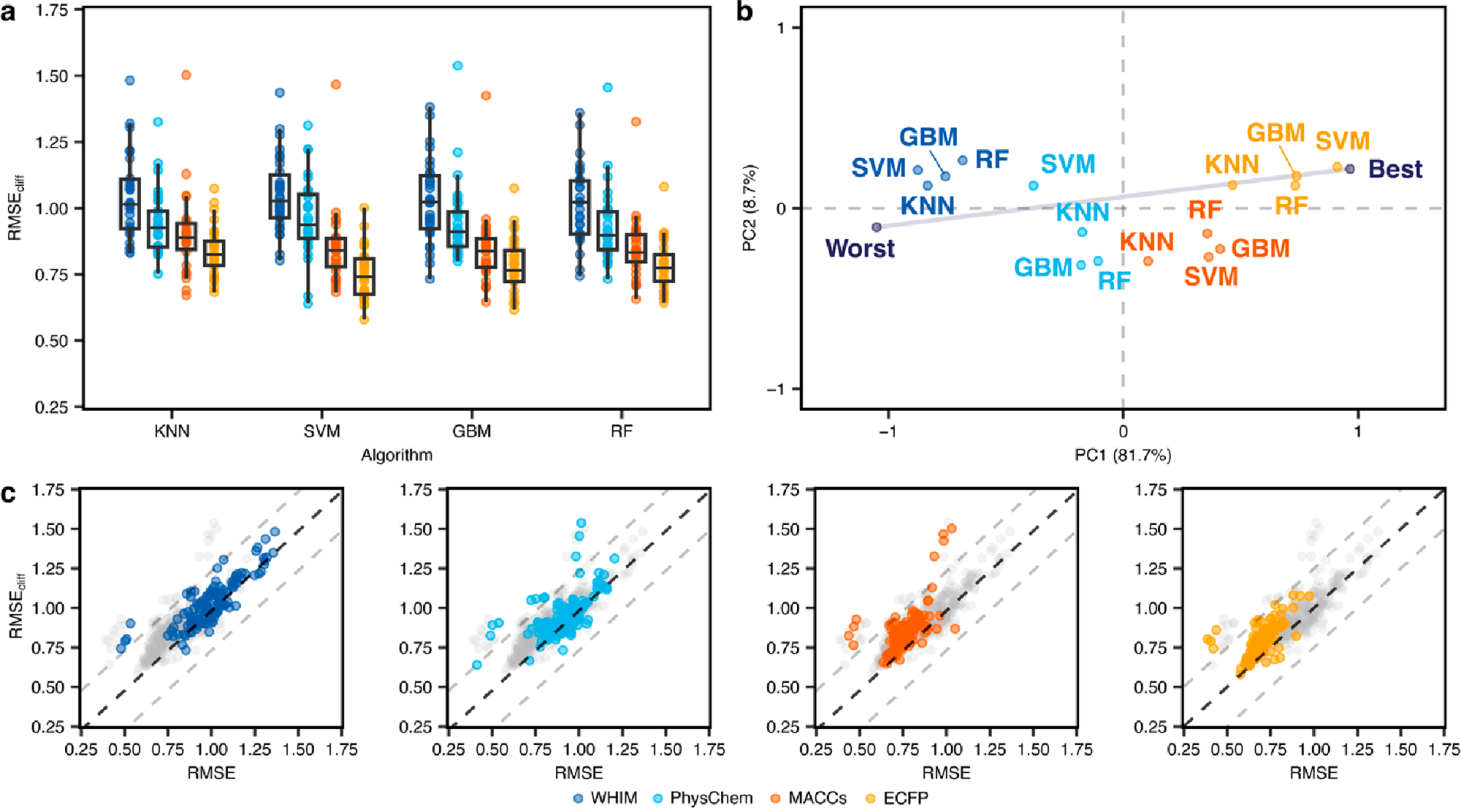
(Corrected) Performance of traditional machine learning methods. (a) RMSE on activity cliff compounds using different machine learning algorithms and molecular descriptors (indicated by colors). (b) Global ranking of all methods using PCA (first two principal components, PC1 and PC2), scaled between best and worst performance. Every point captures a different combination of the machine learning method and the descriptor it relied on and is obtained by considering the corresponding RMSE_cliff_ on all data sets. “Worst” and “Best” indicated the worst and best performance obtained across all data sets, respectively. Percentages represent the variance explained by each principal component. (c) Comparison between the error on activity cliff compounds (RMSE_cliff_) and the error on all compounds (RMSE) for all methods. Black dashed lines indicate RMSE = RMSE_cliff_, while gray dashed lines indicate a difference of ±0.5 log units between RMSE_cliff_ and RMSE.

**Figure 4:**
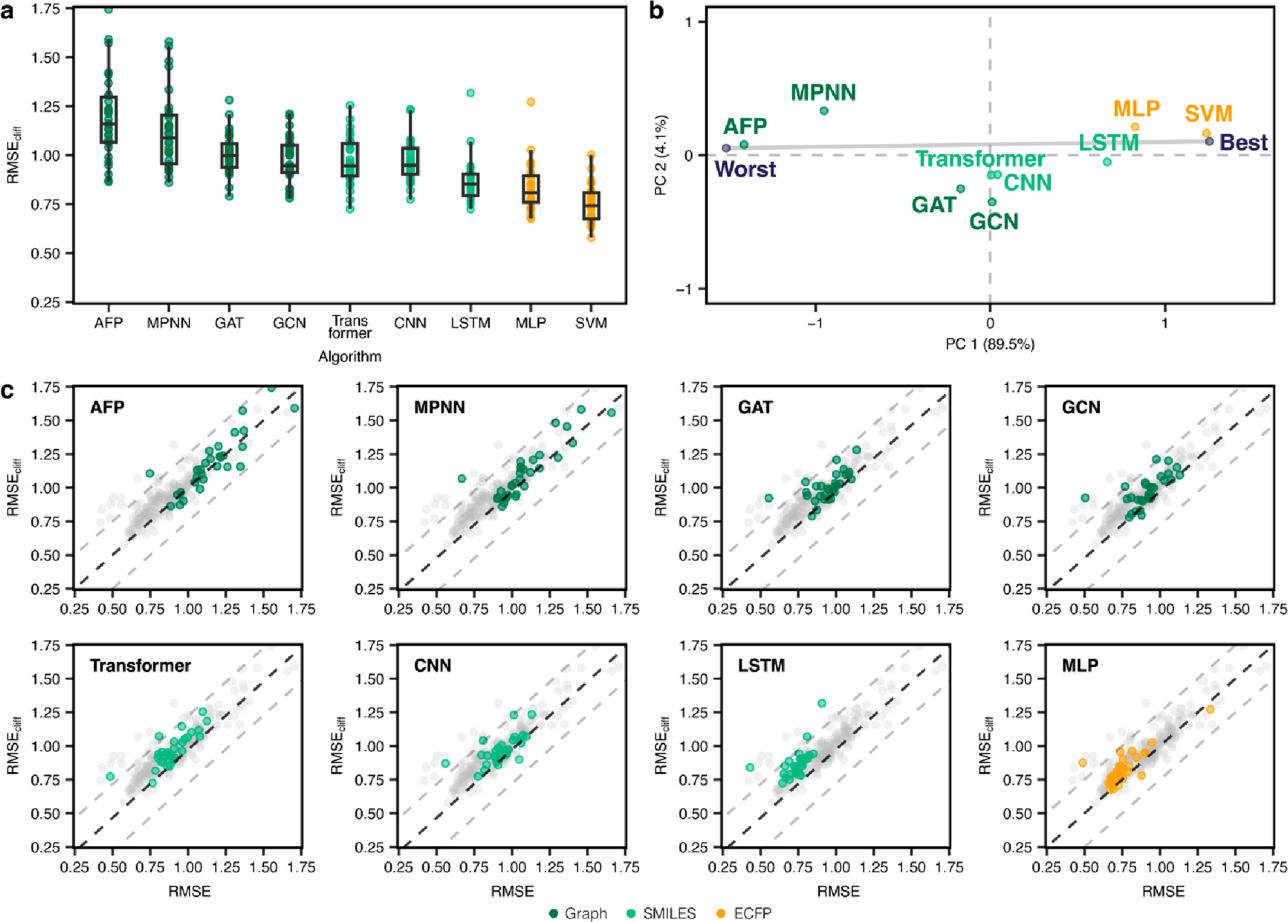
(Corrected) Performance of deep learning methods. (a) RMSE on activity cliff compounds on different deep learning strategies. SVM is reported as a reference. (b) Global ranking of all methods using PCA (first two principal components, PC1 and PC2), scaled between best and worst performance. Every point captures the performance of a different machine learning approach obtained by considering the corresponding RMSE_cliff_on all data sets. “Worst” and “Best” indicated the worst and best performance obtained across all data sets, respectively. Percentages indicate the explained variance by each principal component. (c) Prediction error on activity cliff compounds (RMSE_cliff_) compared to all compounds (RMSE) for all methods.

**Figure 5:**
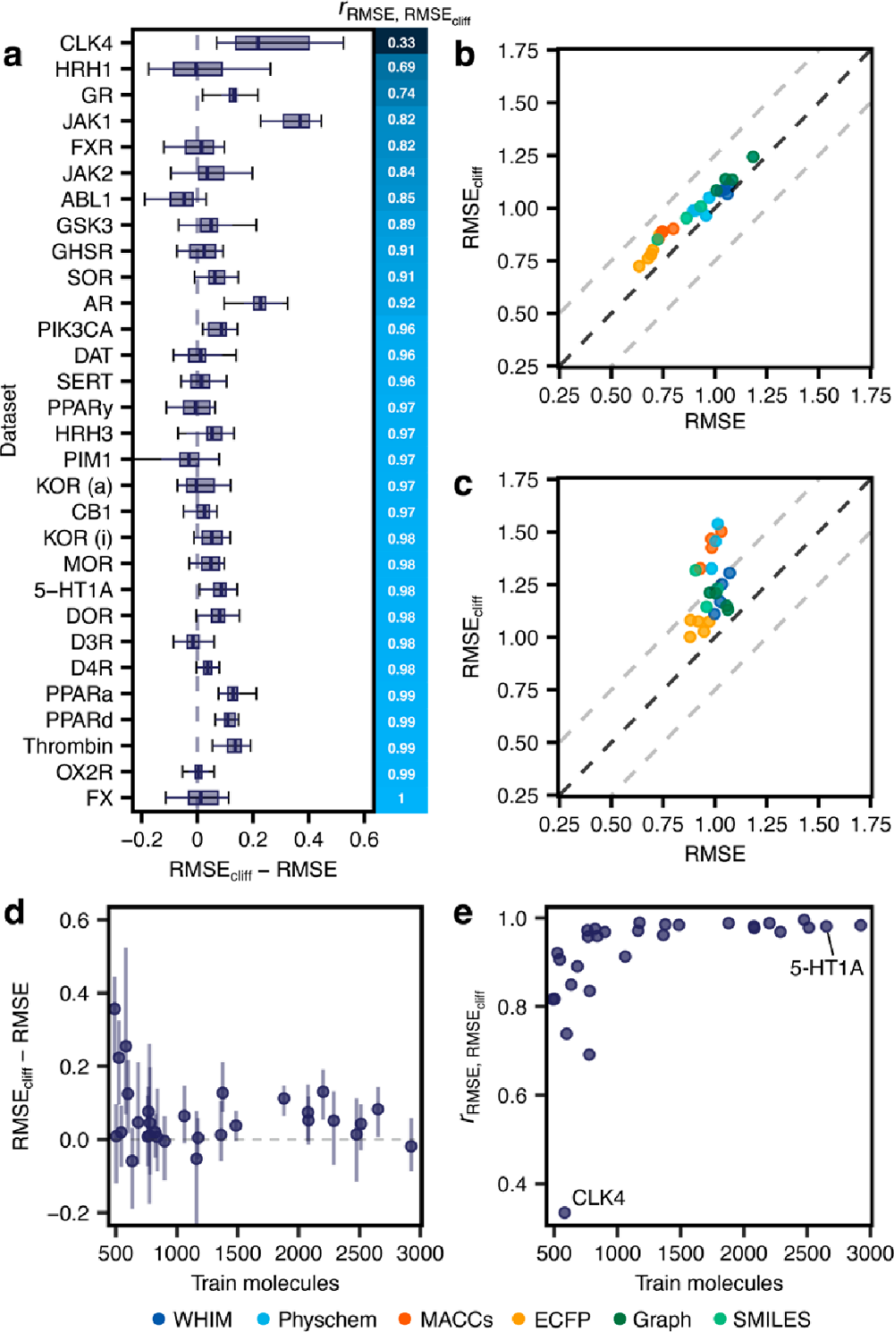
(Corrected) Comparing overall model performance and performance on activity cliff compounds. (a) Method-wide differences between overall RMSE and RMSE_cliff_ for all targets ordered by Pearson correlation (*r*) between RMSE and RMSE_cliff_. Error bars indicate the lowest and highest RMSE_cliff_. (b) Comparison between RMSE and RMSE_cliff_ of all methods on 5-HT1A. (c) Comparison between RMSE and RMSE_cliff_ of all methods on CLK4. (d) Effect of the number of training molecules on the difference between RMSE and RMSE_cliff_. Error bars indicate the lowest and highest RMSE_cliff_. (e) Relationship between the number of training molecules and the Pearson correlation (*r*) of RMSE and RMSE_cliff_.

**Table A:** 

original sentence	updated sentence	page
48,707 molecules (of which 35,632 were unique)	48,714 molecules (of which 35,633 were unique)	5939
7% (JAK1) to 52%	9% (CLK4) to 54%	5940
86.6% of cliff compounds	89.4% of cliff compounds	5940
2.7 ± 0.9 activity cliff partners	3.03 ± 3.34 activity cliff partners	5940
9.1 ± 5.3% of activity cliff molecules	3.5 ± 1.2% of activity cliff molecules	5940
substructure [0.80 ± 0.03]	substructure [0.81 ± 0.09]	5940
scaffold [0.93 ± 0.02]	scaffold [0.92 ± 0.09]	5940
SMILES [0.95 ± 0.01]	SMILES [0.95 ± 0.05]	5940
36,281 molecules	30,048 molecules	5942, 5945
0.62 to 1.60 log units	0.58 to 1.74 log units	5943
0.41 to 1.35 log units	0.39 to 1.91 log units	5943
*r* = 0.81 on average	*r* = 0.91 on average	5944
0.54 log units	0.52 log units	5944
0.094 log units	0.080 log units	5944
0.39 log units	0.32 log units	5944
0.68 to 1.44 log units	0.58 to 1.74 log units	5945
MPNN models resulted in the lowest error on activity cliff compounds on average, although no differences were statistically significant (Wilcoxon rank-sum test with Benjamini–Hochberg correction, Supporting Figure S4).	GCN models resulted in the lowest error on activity cliff compounds on average.	5945
*r* > 0.70 for 25 data sets	*r* > 0.80 for 27 data sets	5945
*r* = −0.15	*r* = −0.17	5945

