# Supplementary material for: Exposing the Limitations of Molecular Machine Learning with Activity Cliffs
Source: J Chem Inf Model. 2022 Dec 1;62(23):5938–51. doi: 10.1021/acs.jcim.2c01073 (PMC9749029; doi:10.1021/acs.jcim.2c01073)
Supplement: Supplementary file 1 — ci2c01073_si_001.pdf [file ci2c01073_si_001.pdf]

Exposing the limitations of molecular machine learning with activity cliffs

Derek van Tilborg,[1,2] Alisa Alenicheva,[3] and Francesca Grisoni[1,2]*

[1]Eindhoven University of Technology, Institute for Complex Molecular Systems and Dept. Biomedical Engineering, Eindhoven, Netherlands.
[2]Centre for Living Technologies, Alliance TU/e, WUR, UU, UMC Utrecht, Utrecht, The Netherlands.
[3]JetBrains Research. Saint Petersburg, Russia.

*Corresponding author: f.grisoni@tue.nl

Table S1 | Presence of highly similar compounds in commercially available libraries. For each library, the number of molecules having *at least* one highly similar neighbor (determined as having a Tanimoto similarity on ECFPs larger than 90%) was reported, along with the corresponding percentage. ECFPs (1024 bits, radius = 2) were computed with RDKit on canonical SMILES strings, after filtering out duplicates and salts, within KNIME 4.3.3.

Provider and library	no. molecules	no. similar	perc. similar
Asinex[a]	572,393	76,794	13.42%
Specs (10, 20, 50 mg)[b]	199,965	13,369	6.69%
Enamine Advanced[c]	604,507	173,383	28.77%
Enamine Premium[c]	40,694	18,936	46.53%

[a]"*All screening compounds*", downloaded from https://www.asinex.com/screening-libraries-(all-libraries) on February 2022.
[b]Downloaded from https://enamine.net/compound-collections/screening-collection/ on February 2022.
[c]Provided by Specs (https://www.specs.net/) on March 2021.

Table S2 | **Dataset overview**, with receptor class, ChEMBL ID, response type (inhibition [K_i] or agonism [EC_{50}]), number of compounds in the train and test set, along with the number of activity cliff compounds in the train and test set.

**Target name (ID)**	**Receptor Class**	**ChEMBL ID**	**Type**	**n train/test**	***n cliff train/test***
Androgen Receptor (AR)	NR	CHEMBL1871	K_i	525/134	126/31
Cannabinoid receptor 1 (CB1)	GPCR	CHEMBL218	EC_{50}	823/208	292/75
Coagulation factor X (FX)	Protease	CHEMBL244	K_i	2476/621	1080/270
Delta opioid receptor (DOR)	GPCR	CHEMBL236	K_i	2077/521	772/193
Dopamine D3 receptor (D3R)	GPCR	CHEMBL234	K_i	2923/734	1150/291
Dopamine D4 receptor (D4R)	GPCR	CHEMBL219	K_i	1485/374	572/143
Dopamine transporter (DAT)	Other	CHEMBL238	K_i	839/213	209/54
Dual specificity protein kinase CLK4	Kinase	CHEMBL4203	K_i	582/149	51/13
Farnesoid X receptor (FXR)	NR	CHEMBL2047	EC_{50}	503/128	195/50
Ghrelin receptor (GHSR)	GPCR	CHEMBL4616	EC_{50}	543/139	262/68
Glucocorticoid receptor (GR)	NR	CHEMBL2034	K_i	598/152	183/47
Glycogen synthase kinase-3 beta (GSK3)	Kinase	CHEMBL262	K_i	683/173	127/31
Histamine H1 receptor (HRH1)	GPCR	CHEMBL231	K_i	776/197	178/46
Histamine H3 receptor (HRH3)	GPCR	CHEMBL264	K_i	2288/574	865/219
Janus kinase 1 (JAK1)	Kinase	CHEMBL2835	K_i	489/126	36/10
Janus kinase 2 (JAK2)	Kinase	CHEMBL2971	K_i	779/197	95/25
Kappa opioid receptor (KOR) agonism	GPCR	CHEMBL237	EC_{50}	762/193	319/81
Kappa opioid receptor (KOR) inhibition	GPCR	CHEMBL237	K_i	2081/521	753/188
µ-opioid receptor (MOR)	GPCR	CHEMBL233	K_i	2512/630	889/222
Orexin receptor 2 (OX2R)	GPCR	CHEMBL4792	K_i	1174/297	610/153
Peroxisome proliferator-activated receptor alpha (PPARα)	NR	CHEMBL239	EC_{50}	1377/344	568/141
Peroxisome proliferator-activated receptor delta (PPARδ)	NR	CHEMBL3979	EC_{50}	900/225	373/94
Peroxisome proliferator-activated receptor gamma (PPARγ)	NR	CHEMBL235	EC_{50}	1879/470	703/178
PI3-kinase p110-alpha subunit (PIK3CA)	Transferase	CHEMBL4005	K_i	767/193	281/70
Serine/threonine-protein kinase PIM1	Kinase	CHEMBL2147	K_i	1162/294	387/98
Serotonin 1a receptor (5-HT1A)	GPCR	CHEMBL214	K_i	2651/666	917/230
Serotonin transporter (SERT)	Other	CHEMBL228	K_i	1362/342	479/120
Sigma opioid receptor (SOR)	Other	CHEMBL287	K_i	1061/267	371/93
Thrombin	Protease	CHEMBL204	K_i	2201/553	790/199
Tyrosine-protein kinase ABL1	Kinase	CHEMBL1862	K_i	633/161	202/51

Table S3 | Activity cliff definitions across different published studies.

**Descriptor**	**Similarity**	***Potency difference***	**Study**
MACCs	Tanimoto coeff. of 0.85	2-fold	Bajorath group. Future Med. Chem. **7**, 1565-1579 (2015); F1000Res, **2** (2013).
ECFPs	Tanimoto coeff. of 0.55	2-fold	Bajorath group. Future Med. Chem. **7**, 1565-1579 (2015); F1000Res, **2** (2013).
MMP	Substructure match	2-fold	Bajorath group. Molecular informatics. **35**, 181-191 (2016); Future Med. Chem. **7**, 1565-1579 (2015); F1000Res, **2** (2013); J. Chem. Inf. Model. **52**, 1138–1145 (2012).
MMP	Substructure match	1 or 2-fold	Pérez-Benito *et al.* J. Chem. Theory Comput. **15**, 1884–1895 (2019).
Several 2D and 3D, including MACCS	Several similarity measures between 0.8 - 0.95	1 or 2-fold	Bajorath group. J. Chem. Inf. Model. **52**, 670–677 (2012).
Maximum common substructure	50% of common substructure atoms	1-fold	Jiménez-Luna. J. Chem. Inf. Model. **62**, 274–283 (2022).
Physicochemical properties	50% of max SALI score	-	Seebeck *et al*. ChemMedChem, **6**, 1630-1639 (2011).
MACCs, ECFP, TARIS, ROCS, Pharmacophore	SALI score	Variable	Medina-Franco *et al. J. Chem. Inf. Model.* ***49****, 477-491 (2009).*
AP descriptor	Dice similarity of 0.7	Custom metric	Sheridan *et al*. J. Chem. Inf. Model. **60**, 1969-1982 (2020).

Table S4 | Training/test set analysis, with number of compounds in the train and test set, the number of activity cliff compounds, the mean number of activity cliff 'partners' per activity cliff compound, the number of activity cliff compounds with all activity cliff 'partners' in the test set, and the mean maximal substructure/scaffold/SMILES similarity of all test activity cliff compounds to the train set.

**Target ID**	**n train/test**	***n cliff train/test***	**Mean cliff partners**	**All cliff partners in test**	***Mean max. similarity test cliff to train***
AR	525/134	126/31	1.66	3	0.78 / 0.89 / 0.96
CB1	823/208	292/75	2.25	3	0.81 / 0.92 / 0.96
FX	2476/621	1080/270	3.25	29	0.83 / 0.94 / 0.96
DOR	2077/521	772/193	2.91	23	0.83 / 0.92 / 0.96
D3R	2923/734	1150/291	2.73	24	0.81 / 0.95 / 0.95
D4R	1485/374	572/143	2.68	12	0.79 / 0.95 / 0.95
DAT	839/213	209/54	1.73	11	0.75 / 0.90 / 0.92
CLK4	582/149	51/13	1.25	0	0.67 / 0.93 / 0.92
FXR	503/128	195/50	2.96	2	0.81 / 0.94 / 0.97
GHSR	543/139	262/68	5.51	0	0.82 / 0.94 / 0.96
GR	598/152	183/47	2.64	9	0.80 / 0.92 / 0.95
GSK3	683/173	127/31	1.59	3	0.78 / 0.92 / 0.93
HRH1	776/197	178/46	1.75	9	0.77 / 0.90 / 0.93
HRH3	2288/574	865/219	2.82	17	0.81 / 0.96 / 0.95
JAK1	489/126	36/10	1.43	0	0.82 / 0.91 / 0.96
JAK2	779/197	95/25	2.3	1	0.77 / 0.95 / 0.94
KOR (a)	762/193	319/81	4.43	4	0.84 / 0.92 / 0.96
KOR (i)	2081/521	753/188	2.99	15	0.83 / 0.92 / 0.96
MOR	2512/630	889/222	3.71	11	0.79 / 0.95 / 0.96
OX2R	1174/297	610/153	2.37	15	0.84 / 0.92 / 0.96
PPARα	1377/344	568/141	2.84	6	0.83 / 0.93 / 0.96
PPARδ	900/225	373/94	2.5	18	0.83 / 0.93 / 0.96
PPARγ	1879/470	703/178	2.18	11	0.80 / 0.95 / 0.95
PIK3CA	767/193	281/70	3.74	6	0.79 / 0.94 / 0.95
PIM1	1162/294	387/98	2.31	21	0.82 / 0.94 / 0.95
5-HT1A	2651/666	917/230	2.16	15	0.80 / 0.94 / 0.94
SERT	1362/342	479/120	2.27	14	0.80 / 0.94 / 0.94
SOR	1061/267	371/93	2.32	17	0.83 / 0.94 / 0.96
Thrombin	2201/553	790/199	3.23	6	0.80 / 0.96 / 0.96
ABL1	633/161	202/51	3.16	18	0.82 / 0.92 / 0.96

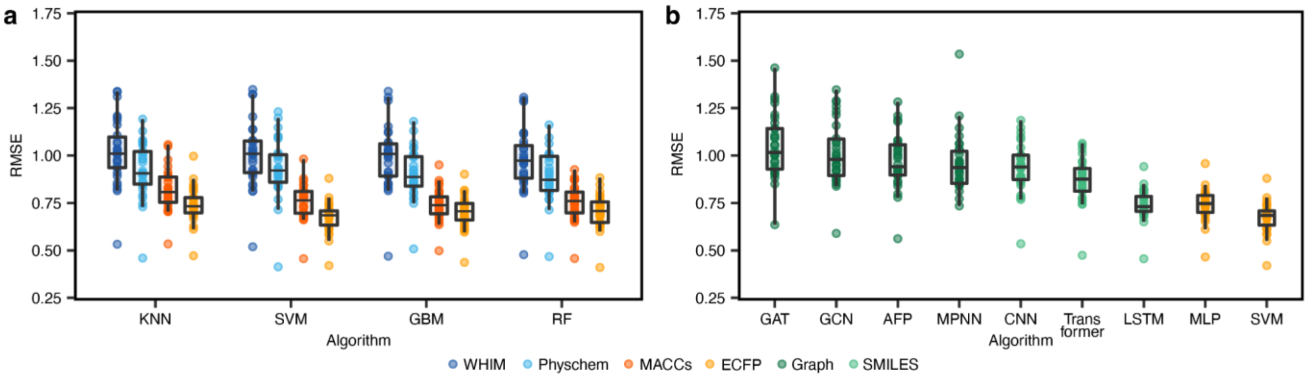

Fig. S1 | **Overall performance of machine learning methods on all targets. a**, RMSE using different traditional machine learning algorithms and molecular descriptors. **b**, RMSE using deep learning methods and unstructured molecular representations.

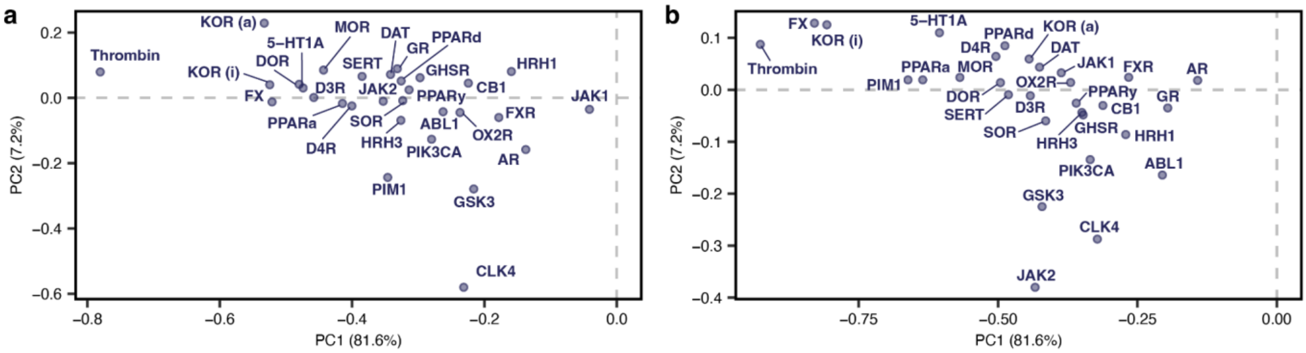

Fig. S2 | **PCA loadings of all methods**. **a**, Effects of individual data sets (loadings for PC1 and PC2) on the PCA of traditional machine learning methods (see Fig. 3b). **b**, Effects of individual data sets (loadings for PC1 and PC2) on the PCA of deep learning methods (see Fig. 4b).

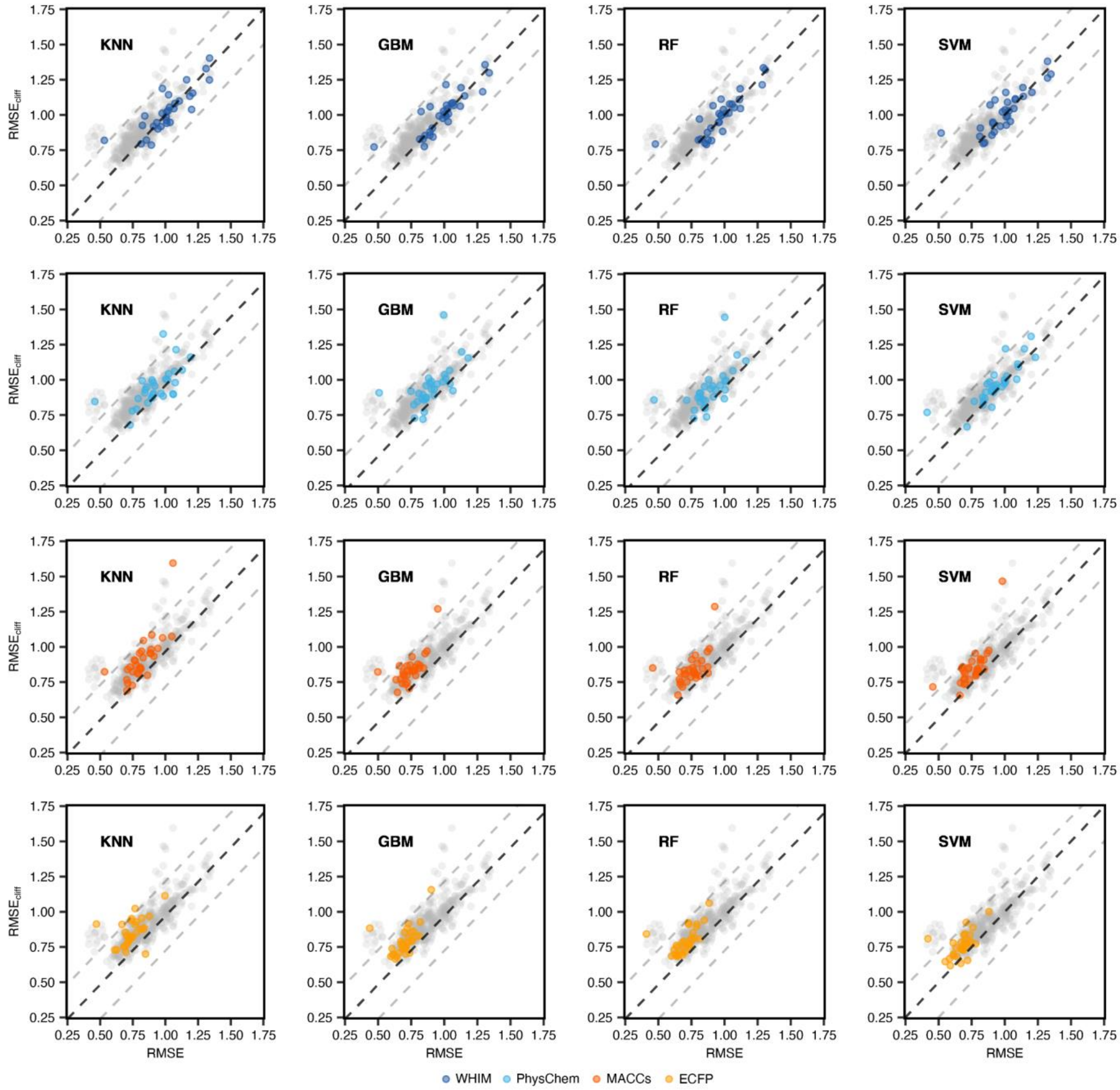

Fig. S3 | Relative prediction error of activity cliff compounds. Prediction error on activity cliff compounds compared to all compounds for all traditional machine learning algorithms and molecular descriptor combinations.

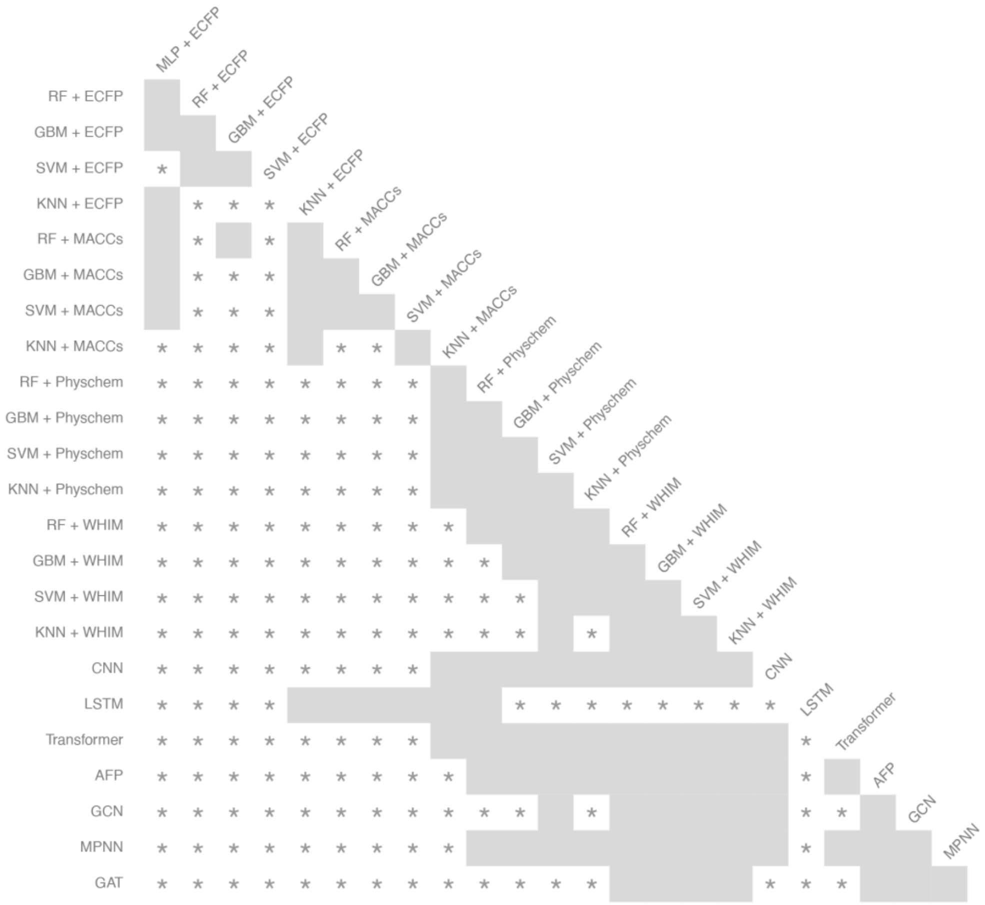

Fig. S4 | Statistical differences between the $RMSE_{cliff}$ values obtained by different machine learning strategies. Asterisks indicate statistically significant differences ($p < 0.05$) between pairs of methods, as obtained by the Wilcoxon rank-sum test (adjusted for false discovery rate using a Benjamini-Hochberg procedure).

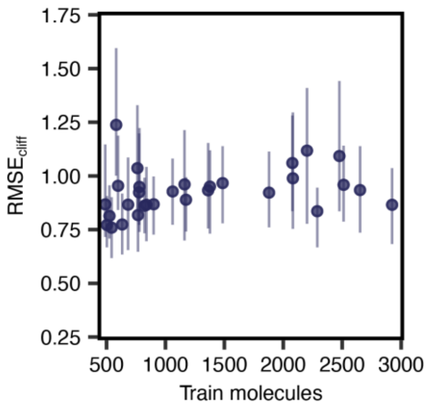

Fig. S5 | Relationship between the number of training molecules on $RMSE_{cliff}$. Error bars indicate the lowest and highest $RMSE_{cliff}$.

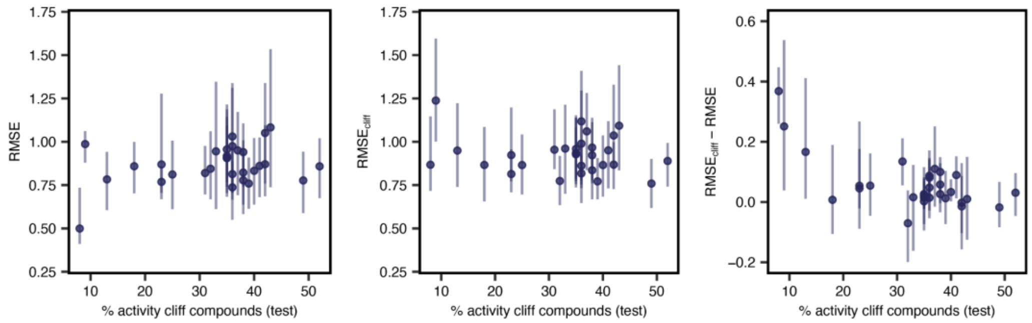

Fig. S6 | Relationship between the fraction of activity cliff compounds and model performance. a, Overall model performance (RMSE). Pearson correlation (*r*) = 0.21. **b**, Performance on activity cliff compounds (RMSE$_{cliff}$, *r*=-0.12). **c**, Difference between RMSE$_{cliff}$ and RMSE (*r*=-0.54). Error bars indicate the lowest and highest RMSEs.

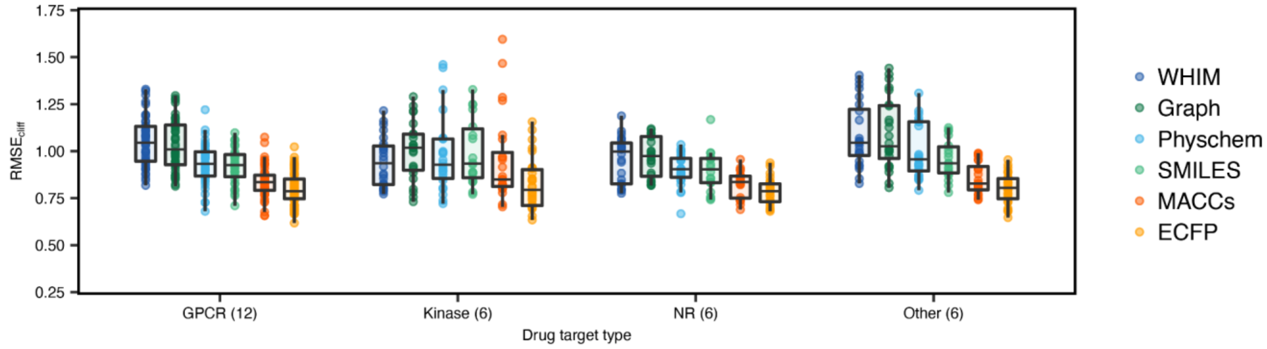

Fig. S7 | Relationship between drug target classes and RMSE$_{cliff}$. All machine learning strategies are grouped by molecular descriptor/representation.
